# The Immune System GTPase GIMAP6 Interacts with the Atg8 Homologue GABARAPL2 and Is Recruited to Autophagosomes 

**DOI:** 10.1371/journal.pone.0077782

**Published:** 2013-10-17

**Authors:** John C. Pascall, Sergio Rotondo, Aamir S. Mukadam, David Oxley, Judith Webster, Simon A. Walker, Jerry Piron, Christine Carter, Nicholas T. Ktistakis, Geoffrey W. Butcher

**Affiliations:** 1 The Babraham Institute, Cambridge, Cambridgeshire, United Kingdom; 2 Laboratory of Lymphocyte Signalling and Development, the Mass Spectrometry Facility, the Babraham Institute, Cambridge, Cambridgeshire, United Kingdom; 3 The Imaging Facility, the Babraham Institute, Cambridge, Cambridgeshire, United Kingdom; 4 The Monoclonal Antibody Unit, the Babraham Institute, Cambridge, Cambridgeshire, United Kingdom; 5 The Inositide Laboratory, the Babraham Institute, Cambridge, Cambridgeshire, United Kingdom; McGill University, Canada

## Abstract

The GIMAPs (GTPases of the **im**munity-**a**ssociated **p**roteins) are a family of small GTPases expressed prominently in the immune systems of mammals and other vertebrates. In mammals, studies of mutant or genetically-modified rodents have indicated important roles for the GIMAP GTPases in the development and survival of lymphocytes. No clear picture has yet emerged, however, of the molecular mechanisms by which they perform their function(s). Using biotin tag-affinity purification we identified a major, and highly specific, interaction between the human cytosolic family member GIMAP6 and GABARAPL2, one of the mammalian homologues of the yeast autophagy protein Atg8. Chemical cross-linking studies performed on Jurkat T cells, which express both GIMAP6 and GABARAPL2 endogenously, indicated that the two proteins in these cells readily associate with one another in the cytosol under normal conditions. The GIMAP6-GABARAPL2 interaction was disrupted by deletion of the last 10 amino acids of GIMAP6. The N-terminal region of GIMAP6, however, which includes a putative Atg8-family interacting motif, was not required. Over-expression of GIMAP6 resulted in increased levels of endogenous GABARAPL2 in cells. After culture of cells in starvation medium, GIMAP6 was found to localise in punctate structures with both GABARAPL2 and the autophagosomal marker MAP1LC3B, indicating that GIMAP6 re-locates to autophagosomes on starvation. Consistent with this finding, we have demonstrated that starvation of Jurkat T cells results in the degradation of GIMAP6. Whilst these findings raise the possibility that the GIMAPs play roles in the regulation of autophagy, we have been unable to demonstrate an effect of GIMAP6 over-expression on autophagic flux.

## Introduction

The GIMAPs are a family of GTPases, occurring sporadically in eukaryotic phyla including molluscs, vertebrates and some protists [1-4]. The family is characterised by the presence of an AIG1 domain (so named after the avrRpt2-induced gene in *Arabidopsis* in which the domain was first identified) which is shared with a family of GTPases in higher plants implicated in the defence response to infection [5]. Sequence analysis has placed the GIMAPs within the TRAFAC class of small GTPases, close to Toc (the translocon at the outer envelope membrane of chloroplasts) and the septins, while structural analysis has additionally revealed features similar to the dynamins [2]. In general, each mammalian species possesses a tight cluster of 7-8 *GIMAP* genes positioned autosomally (chromosome 7q36.1 in humans). The mammalian GIMAP family can be divided into members either with (GIMAP1, 2, 3 and 5) or without (GIMAP4, 6, 7, 8 and 9) predicted transmembrane domains near to their carboxy-termini: none of the members expresses known sequence motifs permitting post-translational lipid modifications, such as prenylation or palmitoylation, that might mediate dynamic membrane associations.

Genetic association studies have implicated *GIMAP* genes in autoimmune diseases *viz*. systemic lupus erythematosus [6,7], Behçet’s disease [8] and type 1 diabetes [9]. Consistent with an involvement in autoimmunity, previous work has implicated the GIMAPs in the control of lymphocyte survival. A spontaneous mutation of *GIMAP5* in rats [10-13], as well as both a mutation and a targeted deletion of *GIMAP5* in mice [14,15], produce severe peripheral lymphopenia in the T lymphocyte lineage. Similarly, a conditional lymphocyte-specific deletion of *GIMAP1* results in severe T lymphopenia; however, in contrast to *GIMAP5* mutants in which B cell numbers are relatively normal in young mice (although they reduce in older animals), these GIMAP1 conditional knockout mice show a profound B lymphopenia even in young animals [16]. The pro-survival activity of these proteins is in contrast to the pro-death activity reported for GIMAP4 in mice [17] and rats [18].

Little is known about the molecular mechanisms by which the GIMAPs influence lymphocyte survival. Findings, indicating that some GIMAP proteins can interact with members of the Bcl-2 protein family [1] and that GIMAP5 may exercise its pro-survival properties by stabilising Mcl-1 [19], suggest that the GIMAPs may provide an extra level of apoptosis regulation special to lymphocytes. 

In order to extend knowledge of the molecular interactions mediating GIMAP function, we have taken a biochemical approach to identifying *in vivo* binding partners for the GIMAPs. Here we present data that (i) identify GABARAPL2 (also known as GATE-16), a mammalian homologue of the yeast autophagy-related gene Atg8, as a major binding partner of GIMAP6 and (ii) demonstrate the relocation of GIMAP6 to autophagosomes in response to cell starvation or mTOR inhibition.

## Materials and Methods

### Materials

Antibodies were sourced from the following companies: anti-MAP1LC3A (product number SAB1408113), anti-MAP1LC3B (L7543), anti-GABARAP (SAB2100873), anti-GABARAPL1 (SAB2103059) anti-SQSTM1 (P0067), and anti-β-ACTIN (A5441) were from Sigma-Aldrich; anti MAP1LC3C (ab150367) was from Abcam; anti-CYCLIN D1 (CC12) was from Calbiochem. Rat monoclonal antibodies to both human GIMAP6 (MAC445) and GABARAPL2 (MAC446) were generated in-house (see below). A rabbit polyclonal antiserum to human GIMAP6 was produced by Harlan Laboratories to an in-house generated antigen (see below).

Inhibitors were from the following sources: PP242 was from Cambridge Bioscience UK; emetine and chloroquine were from Sigma-Aldrich; AZD8055 was a gift from Dr Sylvie Guichard, Astrazeneca UK. G418 (Geneticin) and penicillin/streptomycin were from Invitrogen; all other selective antibiotics used in mammalian cell culture were purchased from InvivoGen.

### Plasmid Constructions

#### a) pcDNA3Biot1His6iresBirA

Initially a biotinylation tag recognition sequence (as described in [20]) was inserted into the multiple cloning site of pcDNA3. Briefly, oligonucleotides JP511 (AGCTTATGTCCGGCCTGAAGACATCTTCGAGGCTCAGAAAAT) and JP512 (GATCCTTCGTGCCATTCGATTTTCTGAGCCTCGAAGATGTCGTTCAGG) were dissolved in water to final concentrations of 10 µM, mixed 1:1 and heated to 95°C in a heating block for 5 minutes. The block was then switched off and the samples cooled to room temperature to allow annealing. The annealed double-stranded oligonucleotide was then ligated between the *Hind*III and *Bam*HI sites of plasmid pcDNA3 to generate pcDNA3Biot1. Subsequently, a DNA fragment spanning the ires-BirA region of a BirA expression vector [20] with flanking *Xho*I and *Xba*I sites was derived by PCR and cloned between the corresponding sites of pcDNA3Biot1 to derive plasmid pcDNA3Biot1iresBirA. This plasmid was further modified by the insertion of a double-stranded oligonucleotide encoding a 6 x His tag with a downstream stop codon between the *Not*I and *Xho*I sites to give plasmid pcDNA3Biot1His6iresBirA.

#### b) Protein expression vectors for transient transfections

 Plasmids biotGIMAP6 and biotGABARAPL2 were derived by PCR-based transfer of human GIMAP6 and GABARAPL2 from full-length cDNA clones BC074744 and BM544477, respectively, into the *EcoR*I-*Xho*I site of pcDNA3Biot1His6iresBirA. Plasmids encoding N-terminally myc-tagged GIMAP proteins or N-terminally HA-tagged proteins were derived by cloning the corresponding cDNA sequences into plasmids pCANmyc1 [21], pCANmyc3 (as pCANmyc1 but with a different reading frame downstream of the myc tag) or pCANHA1 (a derivative of pcDNA3 with an HA tag inserted into the multicloning site), respectively.

#### c) Protein expression vectors for stable cell lines

Plasmid pmycBirA-ires-neo was derived by insertion of a PCR-derived myc-tagged BirA fragment between the *Bam*HI-*Eco*RI sites in plasmid pIRES-neo (Clontech). Plasmid biot-GIMAP6-His-pCAG-iPuro was derived by transfer of a PCR-derived DNA fragment spanning the biotinylation tag-GIMAP6-6His sequence in plasmid biotGIMAP6 into the *Sal*I site of plasmid pCAGiPuro.

### Cell Line Establishment and Maintenance

#### a) Myc-BirA-Jurkat cells

Jurkat T cells were routinely maintained in RPMI/10% fetal calf serum/100 units/ml penicillin/100 µg/ml streptomycin (complete medium). For establishment of myc-BirA-Jurkat cells, approximately 10^7^ Jurkat T cells were transfected by electroporation (260V, 960µF) in RPMI with 20 µg of *Mlu*I-linearized pmycBirA-ires-neo. Cells were allowed to recover overnight in complete medium. The following day, the cells were spun down and resuspended in 20 ml of complete medium and plated at 100 µl/well into 96-well plates. The next day an equal volume of complete medium supplemented with 1 mg/ml (active concentration) G418 (Invitrogen) was added to each well. Every three days thereafter, half of the medium was replaced with fresh complete medium containing 500 µg/ml G418. myc-BirA expressing cell lines were identified by Western blotting (see below). A single myc-BirA-Jurkat cell line was selected for subsequent use.

#### b) Biot-GIMAP6-His myc-BirA Jurkat cell line

The myc-BirA-Jurkat cell line was maintained in complete medium containing 500µg/ml G418. Approximately 10^7^ cells were transfected by electroporation with 20 µg of either *Pvu*I-linearized plasmid biot-GIMAP6-His-pCAG-iPuro or the corresponding vector. Cells were allowed to recover overnight in complete medium containing 500 µg/ml G418. The following day, the cells were spun down and resuspended in 20 ml of complete medium containing 500 µg/ml G418 and plated at 100 µl/well into 96-well plates. The next day an equal volume of complete medium containing 500 µg/ml G418 and 6 µg/ml puromycin was added to each well. Every three days thereafter, half of the medium was replaced with fresh complete medium containing 500 µg/ml G418 and 3 µg/ml puromycin. Cells carrying the parental pCAG-iPuro vector were isolated in parallel, as controls. Biot-GIMAP6-His expressing clones were identified by Western blotting of cell lysates with a horseradish peroxidase-conjugated streptavidin probe. A single vector clone and a single clone carrying Biot-GIMAP6-His (termed the Biot-GIMAP6-His myc-BirA-Jurkat cell line) were maintained for subsequent analysis.

#### c) myc-GIMAP6 HEK293 cell line

HEK293 cells were plated in a 6-well plate and, 24h later, were transfected with a myc-tagged human GIMAP6-expressing plasmid using lipofectamine (Invitrogen). After a further 24 hours, the cells were trypsinised and re-plated in a 10 cm tissue culture plate. 48 hours after transfection, stably transfected cells were selected by growth in DMEM/10% fetal calf serum/100 units/ml penicillin/100 µg/ml streptomycin/800 µg/ml G418. Single colonies were screened for GIMAP6 expression by Western blotting and immunofluorescence. A cell-line expressing a myc-tagged GIMAP6 lacking the C-terminal 10 amino acids was generated similarly.

#### d) Tetracycline-regulated myc-GIMAP6 T-Rex HeLa cell line

An N-terminally myc-tagged GIMAP6-encoding DNA fragment was cloned into plasmid vector pcDNA4.TO and the resulting plasmid transfected into the T-Rex^TM^ HeLa cell line (Invitrogen) by electroporation. Recombinants were selected in DMEM/10% (v/v) FCS/100 units/ml penicillin/100 µg/ml streptomycin containing 5 µg/ml blasticidin S and 100 µg/ml zeocin. Clones transfected with vector plasmid alone were selected in parallel. As required, myc-tagged GIMAP6 expression was induced by addition of tetracycline to a final concentration of 1-2 µg/ml to the culture medium.

#### e) Tetracycline-regulated GIMAP6 shRNA T-Rex Jurkat cells

Complementary oligonucleotide pairs encoding two different shRNA GIMAP6 targets were annealed and cloned between the *Bgl*II and *Hind*III sites of plasmid pTER[22]. The oligonucleotide pairs used were GATCCCGAGTCTAAACTCAGCACCATTCAAGAGATGGTGCTGAGTTTAGACTCTTTTTGGAAA and AGCTTTTCCAAAAAGAGTCTAAACTCAGCACCATCTCTTGAATGGTGCTGAGTTTAGACTCGG (shRNA1) and GATCCCGAACTACAGGAAAGGCAAGTTCAAGAGACTTGCCTTTCCTGTAGTTCTTTTTGGAAA and agcttttccaaaaaGAACTACAGGAAAGGCAAGTCTCTTGAACTTGCCTTTCCTGTAGTTCGG (shRNA2). Resulting plasmid constructs were transfected into the T-REx^TM^ Jurkat cell line (Life Technologies). Recombinants were selected in complete medium containing 10 µg/ml blasticidin S and 200 µg/ml zeocin, and cloned by serial dilution. Cells were treated with 1 µg/ml tetracycline for 4 days to induce shRNA expression prior to analysis.

#### f) HUVEC cells

Primary HUVEC cells purchased from TCS Cellworks were grown in human large vessel endothelial cell basal medium plus human large vessel endothelial cell growth supplement (both from TCS Cellworks) and pencillin/streptomycin.

### Transient Transfection of HEK293T Cells

Cells were maintained in DMEM/10% (v/v) fetal calf serum/penicillin/streptomycin. Transfections were performed using either polyethyleneimine [23] or lipofectamine (Invitrogen) according to the manufacturer’s instructions and cells were analysed 24-48 h later.

### Identification of binding partners for GIMAP6 by formaldehyde-mediated cross-linking and mass spectrometry (MS)

Stably transfected myc-BirA-Jurkat cells carrying either plasmid pCAGiPuro or biot-GIMAP6-His-pCAG-iPuro were grown to approximately 10^6^ cells/ml. Cells (3-6 x 10^8^) were spun down at 400g for 5 min at 4°C, washed with 2 x 20 ml phosphate-buffered saline (PBS), and then resuspended in 20 ml of the same buffer. Formaldehyde solution (37% (w/v) formaldehyde (product number 252549 – Sigma-Aldrich) was added to a final concentration of 1% (w/v), and the cell suspension placed at 20°C for 1h with occasional agitation. The reaction was terminated by the addition of 1/10^th^ volume of 1.25M glycine and left for a further 10 minutes. The cells were then spun down as before, washed once in PBS, and solubilised into 20ml HEPES RIPA lysis buffer (10 mM HEPES, 150 mM sodium chloride, 1.0% (v/v) Triton X-100, 0.1% (w/v) sodium dodecyl sulphate pH 7.5) supplemented with 100 µl mammalian protease inhibitor cocktail (Sigma-Aldrich) at 4°C for 10 minutes. The supernatant was clarified by centrifugation at 20,000 g for 10 minutes at 4°C and passed five times through a streptavidin-agarose affinity column (200 µl packed volume). The column was then washed with 5 x 10 ml of HEPES RIPA buffer and the streptavidin-agarose transferred to a micro-centrifuge tube with 2 x 500 µl of the same buffer. The beads were sedimented at 400 g for 1 minute and then re-suspended in 400 µl of 2 x CSB (160mM Tris-HCl pH6.8, 4% (w/v) sodium dodecyl sulphate, 20% (v/v) glycerol, 200 mM dithiothreitol). The suspension was heated to 100°C for 5 minutes. The beads were then sedimented at 400 g for 1 minute and the supernatant (containing the released proteins) was heated to 100°C to reverse the formaldehyde-induced cross-links.

Protein derived from three separate purifications was pooled, and then precipitated by the addition of trichloroacetic acid to a final concentration of 10% (w/v). Samples were left at 4°C for 20 min and precipitated protein recovered by centrifugation at 20,000 g for 30 min at 4°C. The pellet was washed with 1ml acetone and re-centrifuged as before. The supernatant was aspirated and the pellet dried briefly (30 s) in a 100°C hot block. The pellet was then dissolved in 50 µl 2 x CSB, left at room temperature for 2h and then heated to 100°C for 3 min. Samples were then separated on a 12.5% SDS-PAGE gel and the proteins revealed by staining with Imperial protein stain (Thermo Scientific). Bands of interest were excised and one half of each was reduced, carbamidomethylated and digested overnight with trypsin (Promega sequencing grade, 10 ng/µl in 25 mM ammonium bicarbonate). Approximately 10% of the resulting tryptic digest was analysed by LC-MS/MS. LC separation was achieved on a reversed-phase column (Reprosil C18AQ, 0.075 x 50 mm, 3µm particle size), with an acetonitrile gradient (2 - 35% over 10 min, containing 0.1% formic acid, at a flow rate of 500nl/min). The column was coupled via a nanospray ion source (Proxeon) to a LTQ Orbitrap Velos mass spectrometer (Thermo Scientific) operated in data-dependent acquisition mode. The acquisition cycle consisted of a high resolution precursor ion spectrum over the m/z range 350 - 1500, followed by up to 5 CID spectra. Mass spectrometric data were processed using Proteome Discoverer (Thermo) and searched against the human entries in Uniprot 15.14 using Mascot software (Matrix Science).

### Tag-mediated pull-downs

Approximately 3 x 10^6^ actively growing HEK293T cells were transfected with plasmids as indicated using polyethyleneimine. 24h later cells were transferred to fresh medium. The following day, dishes were washed in PBS and lysed into 1 ml TX100 lysis buffer (10 mM HEPES, 150 mM sodium chloride, 1.0% (v/v) Triton X-100, pH 7.5) supplemented with 100 µl mammalian protease inhibitor cocktail (Sigma-Aldrich) at 4°C for 10 min. The supernatant was clarified by centrifugation at 20,000 g for 10 min at 4°C. An aliquot of the supernatant (50 µl) was removed to an equal volume of 2 x CSB (160 mM Tris pH6.8, 4% (w/v) SDS, 20% (v/v) glycerol, 200 mM dithiothreitol, 0.008% (w/v) bromophenol blue) and boiled to represent a lysate sample. To the remaining supernatant was added either a 50 µl packed volume of streptavidin-agarose (for transfections including pcDNA3Biot1His6iresBirA-based expression plasmids) or 50 µl monoclonal antibody (mAb) 12CA5 (anti-HA) supernatant and a 25 µl packed volume of protein A-Sepharose beads. The suspensions were rotated at 4°C for 4 h, and then centrifuged at 15,000 g for 20 s. The pellets were washed with 8 x 1 ml TX100 lysis buffer and the beads recovered each time by centrifugation at 15,000 g for 20 s. The final washed pellets were resuspended in 100 µl 2 x CSB (and heated to 100°C for 5 min to elute the bound proteins. SDS-PAGE gel electrophoresis and Western blotting of the samples was then performed as described previously [21], using antibodies as indicated in the figures.

### Expression of GST and GST-tagged GIMAP6 and assessment of their *in vitro* interaction with GABARAPL2

A full-length GIMAP6 cDNA fragment was transferred to plasmid pGEX4T-1 and transformed into the *E. coli* Rosetta strain (Novagen). A parallel transformation of plasmid pGEX4T-1 was also performed. 20 mL overnight Rosetta cultures in LB medium containing either the GST or the GST-GIMAP6 expression construct were used to inoculate 1 L pre-warmed LB medium containing 200 μg/mL ampicillin and 50 μg/mL chloramphenicol. Cells were grown at 37 °C for 2-3 h to an OD of approximately 0.6. The cultures were then cooled to 16 °C and protein expression induced by the addition of 0.5 mM IPTG. Cultures were then grown for a further 16 h at 16 °C. All subsequent steps were carried out at 4°C. Bacteria were collected by centrifugation at 5000g for 5 min, pellets washed in 20 mL PBS pH 7.3 and centrifuged as described above. The pellets were then re-suspended in 20 mL lysis buffer (PBS containing 5mM EGTA, 1% (v/v) Triton-X100, 5mM MgCl_2_, pH 7.4) per pellet and the cells lysed by sonication. The resulting lysate was centrifuged at 10000 g for 15 min and the supernatant applied to a 125 µl packed volume glutathione Sepharose 4B column previously pre-equilibrated in binding buffer (140 mM NaCl, 2.7 mM KCl, 1mM DTT, 10 mM Na_2_HPO_4_, 10 mM KH_2_PO_4_ pH7.3. The flow-through was collected and rebound to the column twice more. The column was washed with 3 x 5 column volumes of binding buffer. Purified GABARAPL2 (20 µg) in 500 μL of binding buffer was added to the column and incubated for 1 h at 4 °C. The column was washed as detailed above, and the bound protein (GST and GST-GIMAP6) was eluted by adding 2 x 100 µl of elution buffer (50 mM Tris-HCL, 10 mM reduced glutathione, pH 8.0). Eluates were analysed by SDS-PAGE, and were then either stained with Coomassie Brilliant Blue R to assess recovery of GST/GST-GIMAP6 or by Western blotting to assay for the presence of GABARAPL2.

### Generation of monoclonal antibodies

Monoclonal rat IgG antibodies raised to full-length human GIMAP6 and GABARAPL2 fused to glutathione-S-transferase were prepared as described previously [21], except that inclusion body extracts in 4-6M urea were dialysed against PBS prior to injection without further purification. Animal procedures were carried out in strict accordance with EU and UK Home Office Regulations (Project Licence No. PPL80/2359), and with the approval of the Babraham Institute Animal Welfare, Experimentation, and Ethics Committee. Rabbit polyclonal antiserum was also raised to the same GIMAP6 antigen by Harlan Laboratories, Loughborough, UK. Specificities of the antibodies and antiserum were tested against over-expressed myc-tagged GIMAP and Atg8 family proteins generated in HEK293T cells (Figure S1: monoclonal antibody MAC445- anti-GIMAP6 and rabbit anti-GIMAP6 polyclonal antisera; Figure S2: monoclonal antibody MAC446 – anti-GABARAPL2).

### Site-directed mutagenesis

Site-directed mutagenesis was routinely performed using a PCR-based strategy with the Accuzyme DNA polymerase (Bioline). N- and C-terminally truncated fragments were generated using oligonucleotides located at the desired ends of the fragments. Internal site-specific mutations were introduced by generating two overlapping PCR products incorporating the desired mutation within the overlapping region. These were then joined in a second PCR reaction spanning the whole desired fragment. The inserts of all PCR-derived plasmids were sequenced completely to ensure that no unwanted mutations had been introduced.

### Induction of autophagy by cell starvation or drug treatment

Cells to be starved were washed twice with starvation medium (140mM NaCl, 1mM CaCl_2_, 1mM MgCl_2_, 5.5mM glucose, 20 mM HEPES pH 7.4, 1% (w/v) BSA) and then maintained in the same medium for the duration of the starvation. In some experiments, mTOR inhibitors AZD8055 or PP242 were added directly to cells in complete growth medium to final concentrations of 1 µM or 0.45 µM, respectively, to induce autophagy.

### Analysis of the levels of RNA species by qPCR

Total RNA was isolated from myc-GIMAP6 T-Rex HeLa cells, either treated with 1 µg/ml tetracycline for 4 days or left untreated, using RNeasy columns (Qiagen) as described by the manufacturer. cDNA synthesis was performed using a Quantitect reverse transcription kit (Qiagen) and then qPCR performed on the resulting cDNA using a SYBR Green PCR master mix (Applied Biosystems) in a Biorad CFX-96 Real Time system with the following oligonucleotide pairs: GAPDH – AATGAAGGGGTCATTGATGG and AAGGTGAAGGTCGGAGTCAA; MAP1LC3B – AAGCTGCTTCTCACCCTTGT and GAGAAGACCTTCAAGCAGCG; SQSTM1 – TTCTTTTCCCTCCGTGCTC and GGATCCGAGTGTGAATTTCC ; GABARAPL2 – GTGTTCTCTCCGCTGTAGGC and AGGCGATCTTCCTGTTTGTG; GIMAP6 – Gactgtcccagatccagaag and GCTGGCAACTAGAAGGCACA. It should be noted that as the myc -GIMAP6 DNA sequence in these cells does not contain an intron, qPCR was performed between the GIMAP6 sequence and the downstream bovine growth hormone splice/polyadenylation sequence to ensure a size difference between the integrated cDNA fragment and the spliced mRNA.

### Determination of GABARAPL2 half-life

HEK-293 cells engineered to overexpress myc-GIMAP6, or cells carrying the corresponding vector, were grown on 35 mm dishes. Emetine was added to the medium to a final concentration of 10 µMDishes were incubated in the presence of the inhibitor for various times as indicated. Cells were then washed with 2 ml PBS, lysed into 100 µl CSB and boiled for 3 min. Aliquots (20 µl) were then resolved by SDS gel electrophoresis and analysed by Western blotting. 

### Immunocytochemical localization of proteins in cell lines

Cells grown on glass coverslips were washed with PBS and then fixed by one of two methods:-

a. Cells were fixed in 4% (w/v) paraformaldehyde for 20 min at room temperature. Following fixation, coverslips were rinsed 2-3 times with PBS and left shaking gently for 15 min to remove all traces of paraformaldehyde before subsequent processing.
b. Cells were fixed in ice-cold methanol for 1-2 min and then in 1% (w/v) bovine serum albumin in PBS with shaking at room temperature for at least 1 h.

For studies on Jurkat T cells, coverslips were first treated with 0.1% (w/v) poly-L-lysine to promote cell attachment prior to cell growth and were then fixed in paraformaldehyde as indicated above.

Sections were subsequently processed as described previously [24] except that DMEM/10% (v/v) FCS was replaced by PBS containing 1% (w/v) BSA throughout the protocol, the incubation times for primary and secondary antibodies were reduced to 30 min and Triton-X100 permeabilisation was omitted for methanol-fixed cells. Cells were finally mounted using Aqua Poly Mount (Polysciences Inc.). Confocal images were captured using an Olympus FV1000 imaging system. Co-localisation of proteins was determined using Imaris software. For immunocytochemistry of HUVEC cells, fluorescence microscopy was performed using an Axio Imager.D2 microscope (Carl Zeiss Microscopy), and for Jurkat T cells, images were captured using a Nikon N-SIM super-resolution system with a CFI Apo TIRF 100x oil objective lens (N.A. 1.49).

## Results

### GABARAPL2 is a binding partner of human GIMAP6

**Figure 1:**
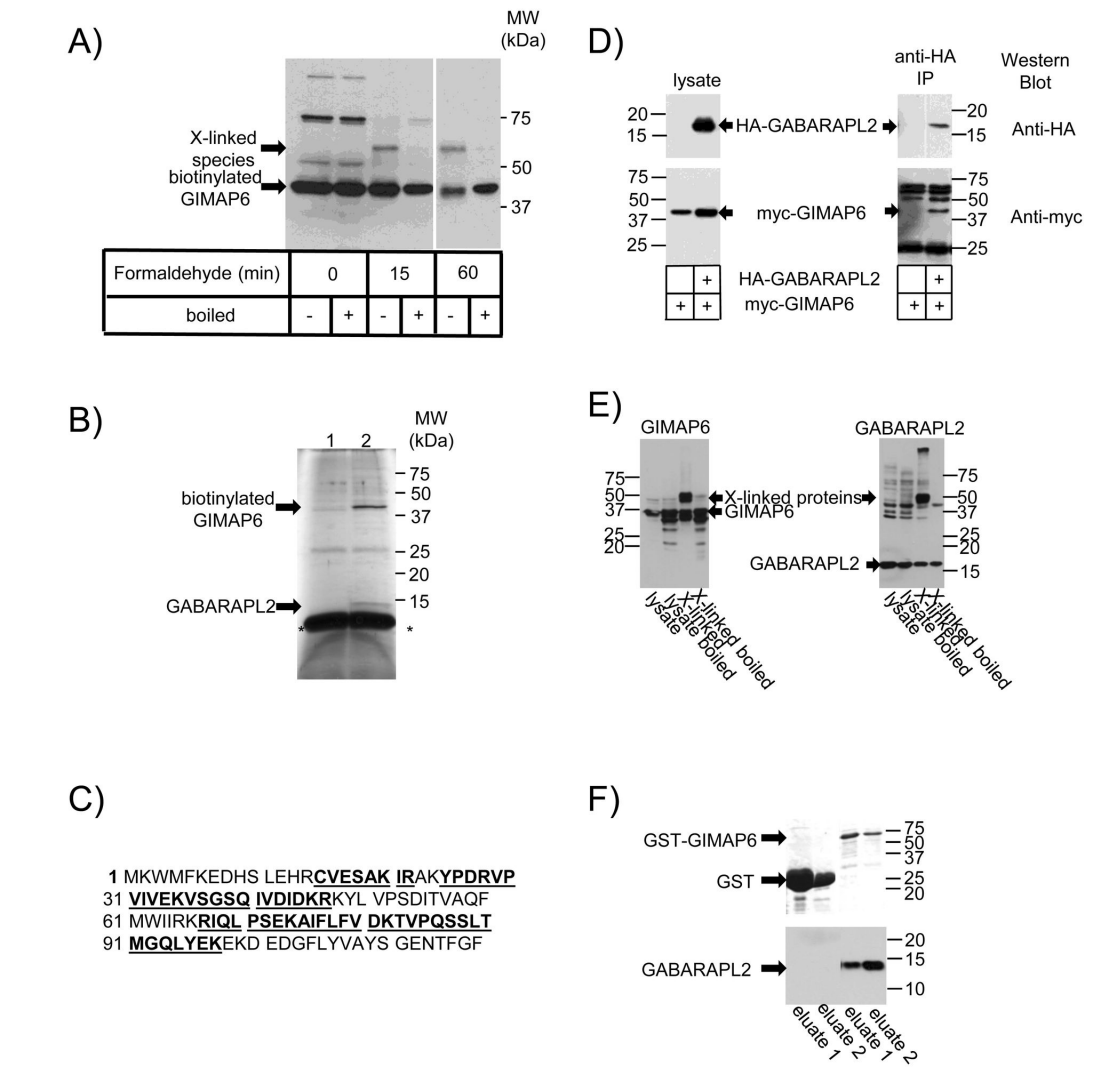
GIMAP6 interacts with GABARAPL2 in mammalian cells and *in vitro*. A) Jurkat T-cells engineered to over-express myc-tagged BirA and GIMAP6 carrying a biotinylation target sequence were incubated with or without 1% (v/v) formaldehyde at room temperature for the indicated times. Cell lysates were analysed by Western blotting with a streptavidin-HRP conjugate to reveal biotinylated proteins. The mobilities of biotinylated GIMAP6 and the cross-linked species (X-linked species) are shown. The electrophoretic mobility of molecular weight protein standards run in parallel is indicated. Note that the gap between the 15 and 60 min samples indicates that intermediate tracks have been removed. B) Silver-stained SDS-PAGE gel of purified biotinylated GIMAP6 and co-purifying proteins from myc-birA Jurkat cells stably transfected with either plasmid biot-GIMAP6-His-pCAG-iPuro (Lane 2) or the corresponding vector (Lane 1). The electrophoretic mobilities of biotinylated human GIMAP6 and GABARAPL2 are shown, as are those of molecular weight protein standards run in parallel. The asterisks (*) indicate the location of streptavidin released from the streptavidin-agarose beads. C) The sequence of GABARAPL2. Sequence coverage detected in tryptic peptides by the mass spectrometry analysis are shown in bold and underlined. D) HEK293T cells (3 x 10^6^) were transfected with a plasmid (10 µg) encoding myc-tagged human GIMAP6 together with a plasmid (10 µg) encoding either an HA-tagged GABARAPL2 or the corresponding vector. Post-nuclear supernatants were then either directly separated by SDS-PAGE (lysate) or immunoprecipitated with anti-HA mouse mAb 12CA5 and protein-A Sepharose prior to SDS-PAGE (anti-HA IP). Separated proteins were analysed by Western blotting using either anti-HA mouse mAb 12CA5 or anti-myc mAb 9E10 followed by HRP-conjugated goat anti-mouse IgG. E) Jurkat T cells (approximately 9 x 10^7^) were incubated in PBS with or without 1% (w/v) formaldehyde for 1 h at room temperature. The reaction was terminated by adding 1/10^th^ volume of 1.25 M glycine, and cells solubilised into 200 µl TX100 lysis buffer containing mammalian protease inhibitors (Sigma). After centrifugation (20000 g, 5 min, 4°C) an equal volume of 2 x CSB was added and the samples either heated at 100°C (boiled) for 30 min to reverse the formaldehyde-induced cross-links or left untreated. Aliquots of the boiled and unboiled (untreated or cross-linked) lysates were then separated on SDS-PAGE gels and then Western blotted, using rat mAb MAC445 to human GIMAP6 - (left panel) or rat mAb MAC446 to GABARAPL2 (right panel) followed by horse-radish peroxidase (HRP) conjugated goat F(ab’)_2_ fragment anti-rat IgG. Blots were then developed using Immobilon ECL western blotting substrate. Cross-linked samples are indicated by “X-linked”. The mobilities of GIMAP6 and GABARAPL2 are indicated. F) Glutathione Sepharose 4B-immobilised GST or GST-GIMAP6 was incubated with bacterially expressed purified GABARAPL2. Proteins were then eluted with glutathione and the eluates (two eluted fractions from each column), resolved by SDS-PAGE. Proteins were then visualised either by Coomassie Blue staining or by Western blotting with anti-GABARAPL2 monoclonal antibody MAC446. Results shown in panels A, D, E and F are representative of data obtained in at least two independent experiments. Panel B shows a typical example of one of the three pooled purifications performed to allow identification of GIMAP6-interacting proteins.

In a search for molecular binding partners for GIMAP proteins, we performed streptavidin affinity purifications from *in vitro* transfected cells (HEK293T, Jurkat-T) engineered to express both the biotinylating enzyme BirA and a GIMAP construct incorporating a target sequence for this enzyme [20]. In some experiments, we combined this approach with formaldehyde cross-linking in order to stabilise possible molecular interactions. For several GIMAPs this strategy was unsuccessful but, in the case of GIMAP6, a discrete formaldehyde cross-linked species was obtained that exhibited slower mobility in SDS-PAGE than biotinylated GIMAP6 - M_r_ ca. 55,000 compared with ca. 40,000 for biotinylated GIMAP6, suggesting cross-linking of GIMAP6 to a second protein species of approximately 15kD. This 55K band was lost after disruption of cross-links by boiling (Figure 1A). Consistent with complex formation between the biotinylated GIMAP6 and an additional protein, in experiments in which cross-linking was omitted, an *M*_r_ ~15K band was observed to co-purify with biotinylated GIMAP6 after streptavidin-affinity purification, although this band was at a much lower relative intensity with respect to the biotinylated GIMAP6 band (Figure S3) than the 55K species observed after cross-linking.

By scaling up the cross-linking protocol using the Biot-GIMAP6-His myc-BirA-Jurkat cell line (see Materials and Methods section), we were able to prepare sufficient of the ~15K band to perform mass spectrometric analysis (Figure 1B). On the evidence of nine distinct tryptic peptides, the band was identified as GABARAPL2 (predicted molecular weight [unmodified] =13,667) (Figure 1C), one of seven human homologues of the yeast Atg8 protein [25]. The peptides identified covered approximately half of the known polypeptide sequence of mature GABARAPL2. 

To confirm the observed interaction, the ability of human GIMAP6 and GABARAPL2 to interact when transiently over-expressed in HEK293T cells was investigated. When HA-tagged GABARAPL2 was immunoprecipitated with an anti-HA mAb, co-transfected myc-tagged GIMAP6 was also recovered in the immunoprecipitated material (Figure 1D). However, the myc-tagged protein was not observed in immunoprecipitates prepared from cells from which HA-tagged GABARAPL2 was absent.

As Jurkat-T cells express GIMAP proteins endogenously [24,26], we used these cells to address the question of whether an interaction could be detected between GIMAP6 and GABARAPL2 when expressed at endogenous levels. Using novel monoclonal antibodies that we prepared to the two proteins, we found that, after cross-linking, a proportion of the material immunoreactive with our anti-human GIMAP6 antibody in Western blotting acquired a slower mobility (Figure 1E). The apparent size shift (~37K to ~51K) was consistent with the earlier experiments using the internally biotinylated GIMAP6 construct as well as with the molecular weight of GABARAPL2. Probing of a second Western blot run in parallel with an antibody against GABARAPL2 revealed a cross-linked product of the same size, suggesting strongly that this corresponds to the same complex. Interestingly, the principal complex formed by GABARAPL2 appears to be that with GIMAP6, although some material is cross-linked into a higher molecular weight complex(es). Unfortunately, we have not been able to demonstrate directly an interaction between the two endogenous proteins by co-immunoprecipitation (Figure S4A). This could be due to the inability of our antibodies to recognise the native complex *in vivo* or could reflect the transient nature of the interaction between the proteins expressed at endogenous levels, which is captured by cross-linking but which is too brief to survive a normal immunoprecipitation protocol. As GABARAPL2 has been shown to have key roles in autophagy (see below), we investigated the possibility that induction of autophagy might allow us to detect GIMAP6-GABARAPL2 interaction. However, although starvation of Jurkat-T cells induced autophagy, as assessed by the formation of MAP1LC3B-II from MAP1LC3B-I (Figure S4B), we were still unable to show co-immunoprecipitation of GABARAPL2 with GIMAP6 ( Figure S4A). 

To address the issue of whether the interaction between GIMAP6 and GABARAPL2 was direct or required additional proteins, glutathione-S-transferase (GST) or a GST-GIMAP6 fusion protein were expressed in *Escherichia coli* and the two proteins purified on glutathione beads. The beads carrying the proteins were then incubated with bacterially expressed purified GABARAPL2 (a kind gift from Michael Wilson, The Babraham Institute). After extensive washing, the GST proteins were eluted from the beads with glutathione and the eluates assessed for the presence of associated GABARAPL2. Although GST was expressed at much higher levels than the GST-GIMAP6 fusion protein, whereas no GABARAPL2 was found to elute with GST, it was detected in the GST-GIMAP6 eluates (Figure 1F), indicating that the interaction between GIMAP6 and GABARAPL2 is direct and does not require other proteins.

### Specificity of the GIMAP6-GABARAPL2 interaction

**Figure 2:**
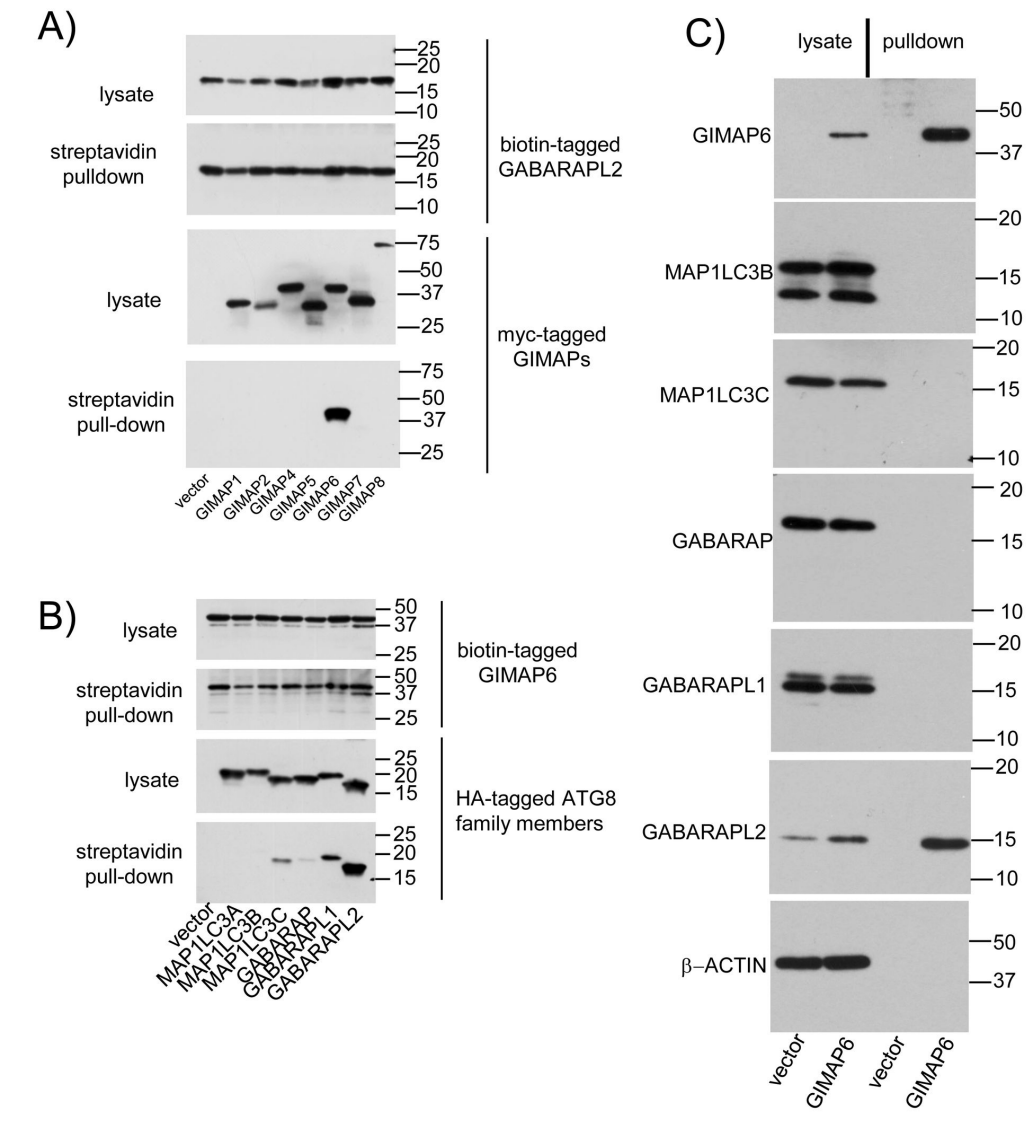
Specificity of GIMAP6-GABARAPL2 interactions. A) HEK293T cells were transfected with 10 µg GABARAPL2 in pcDNA3Biot1His6iresBirA and 10 µg myc-tagged human GIMAP-encoding plasmids as indicated. 48 h later, cell lysates were prepared and biotinylated GABARAPL2 and associated proteins recovered on streptavidin-agarose beads. Aliquots of the lysates and recovered proteins were separated by SDS-PAGE and blotted for biotinylated proteins using HRP-conjugated streptavidin or myc-tagged proteins using mouse anti-myc mAb 9E10 followed by HRP-conjugated goat anti-mouse IgG. B) HEK293T cells were transfected with 10 µg human GIMAP6 in pcDNA3Biot1His6iresBirA and 10 µg HA-tagged Atg8-encoding plasmids as indicated. 48 h later, cell lysates were prepared and biotinylated GIMAP6 and associated proteins recovered on streptavidin-agarose beads. Aliquots of the lysates and recovered proteins were separated by SDS-PAGE and blotted for biotinylated proteins using HRP-conjugated streptavidin or HA-tagged proteins using mouse anti-HA mAb 12CA5 followed by HRP-conjugated goat anti-mouse IgG. Blots were developed using Immobilon ECL western blotting substrate. C) Biotinylated proteins were purified from the Biot-GIMAP6-His myc-BirA-Jurkat cell line or the corresponding vector-only cell line using streptavidin-agarose. Western blots of lysates prepared directly from the cells or of the purified proteins were then probed with HRP-conjugated streptavidin to visualise biotinylated GIMAP6 or with MAb MAC446 to GABARAPL2 or antibodies (as detailed in the Materials and Methods section) to other Atg8 family members or β-ACTIN as described by the suppliers. Results in all three panels are representative of data obtained from at least two independent experiments.

Both GIMAP6 and GABARAPL2 are members of multigene families and, in both cases, individual members of these families are predicted to perform related functions. It was therefore important to determine the selectivity of the observed interaction. Using pull-downs from co-transfected HEK293T cell lysates, we first tested the ability of GABARAPL2 to discriminate between mammalian GIMAP family members. Biotin-tagged human GABARAPL2 proved to be highly selective for GIMAP6 amongst a panel of all of the human GIMAP proteins (Figure 2A). Next we tested the ability of biotin-tagged GIMAP6 to pull down HA-tagged human Atg8 family members (Figure 2B), namely MAP1LC3A (alternative names LC3A/LC3-α), MAP1LC3B (alternative names LC3B/LC3-β), MAP1LC3C (alternative names LC3C/LC3-γ), GABARAP, GABARAPL1, and GABARAPL2 (GATE-16). In this case, whilst GIMAP6 interacted strongly with GABARAPL2, interactions detectable with other family members (MAP1LC3-γ, GABARAP and GABARAPL1) were much weaker. 

As in this system both the GIMAP and Atg8 family members were over-expressed, we considered the possibility that the weaker interactions observed were being driven by this, rather than reflecting the *in vivo* situation. Although, as discussed above, endogenous GABARAPL2 could not be co-immunoprecipitated with GIMAP6, preliminary results indicated that endogenous GABARAPL2 could be co-purified with biotinylated GIMAP6 by streptavidin-affinity chromatography from the Biot-GIMAP6-His myc-BirA-Jurkat cell line. We therefore used a panel of antibodies raised to members of the human Atg8 family to determine whether they too could be co-immunoprecipitated. The specificity of all of the antibodies used was first tested against lysates of a panel of HA-tagged Atg8 members transiently over-expressed in HEK293T cells. The antibodies to MAP1LC3A, MAP1LC3C, and GABARAPL2 (the latter prepared in house – see later) reacted only with their expected targets. In contrast, the antibody to MAP1LC3B also reacted with MAP1LC3A, as reported previously [27], and antibodies to GABARAP and GABARAPL1 reacted with both proteins (Figure S2). 

Using this panel of antibodies, Western blot analysis of proteins co-purifying with biotinylated GIMAP6 from the Biot-GIMAP6-His myc-BirA-Jurkat cell line indicated that, of the Atg8 family members we could detect in cell lysates (all except MAP1LC3A), only GABARAPL2 co-purified with GIMAP6 (Figure 2C), thus providing further evidence that the GIMAP6-GABARAPL2 interaction is much preferred over interaction with other family members.

### Molecular requirements for the GIMAP6-GABARAPL2 interaction

**Figure 3:**
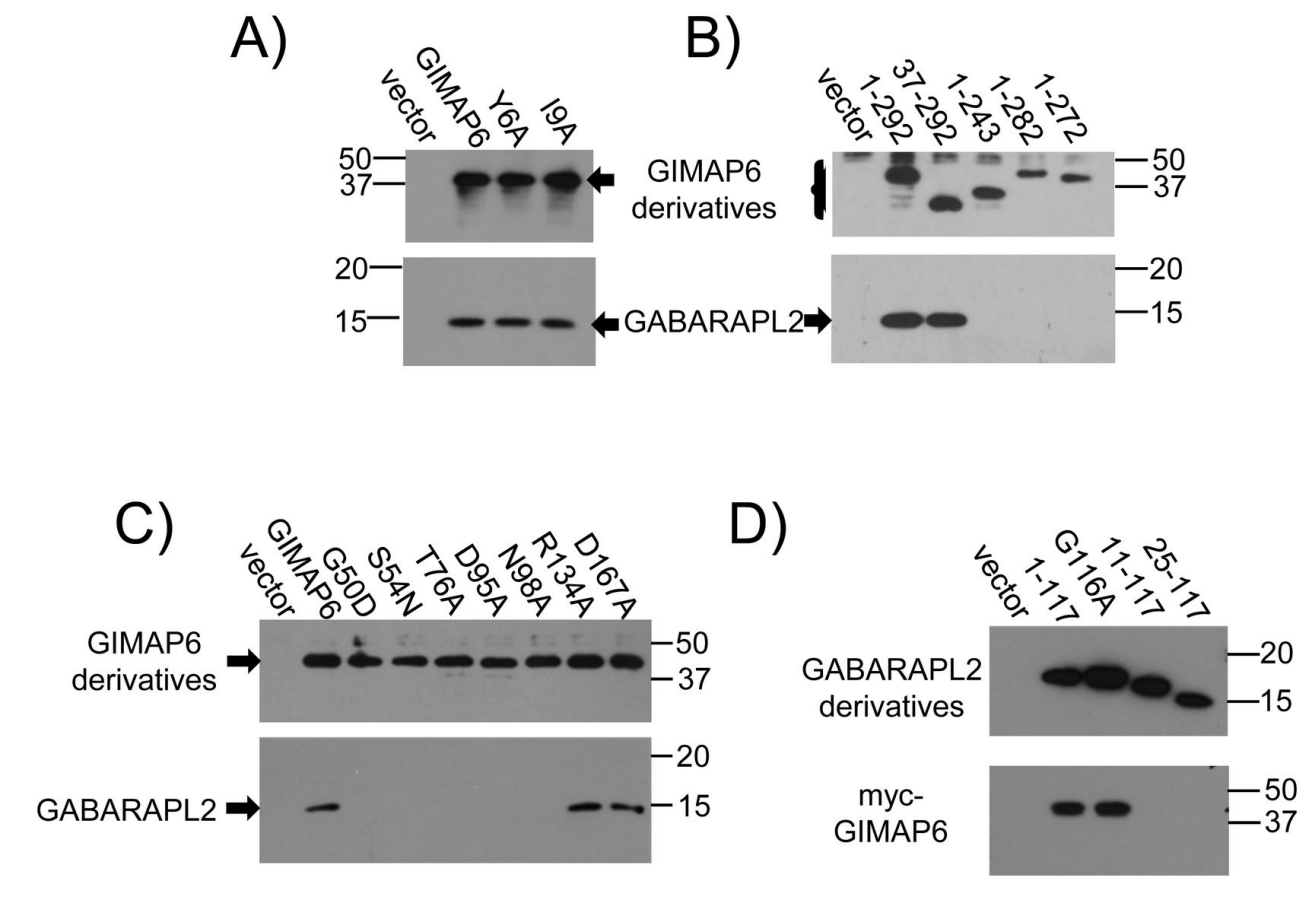
Identification of the domains of GIMAP6 and GABARAPL2 required for their interaction. Panels A-C) HEK293T cells were transfected with 10µg wild-type GIMAP6 or the indicated mutated derivatives in plasmid pcDNA3Biot1His6iresBirA. Biotinylated and associated proteins were recovered by streptavidin-agarose affinity chromatography 48 h after transfection. Western blots of the recovered proteins were probed with HRP-conjugated streptavidin (to show the GIMAP6 proteins) or rat monoclonal antibody MAC446 to GABARAPL2 followed by an HRP-conjugated goat F(ab’)_2_ fragment anti-rat IgG. Panel A) GIMAP6 compared with mutations of the putative AIM motif in GIMAP6. Panel B) GIMAP6 compared with N- and C-terminal mutants of the protein as indicated. In panel B, 1-292 corresponds to full-length GIMAP6. Panel C) Mutations within the GTPase domain of GIMAP6 as indicated. Panel D) HEK293T cells were transfected with a plasmid encoding myc-GIMAP6 together with plasmids encoding biotinylated forms of GABARAPL2 as indicated. Cell lysates were prepared and biotinylated and associated proteins recovered by streptavidin-agarose affinity chromatography. Western blots were probed with HRP-conjugated streptavidin (to show the GABARAPL2 proteins) or an anti-myc monoclonal antibody 9E10 followed by an HRP-conjugated goat anti-mouse IgG to detect myc-tagged GIMAP6. The wild-type protein is represented by 1-117. Results in all four panels are representative of data obtained from three independent experiments.

Commonly, proteins that interact with members of the Atg8 family carry a characteristic Atg8-family interacting motif (AIM), sometimes termed an LC3-interacting region (LIR) (see 28 for review). Inspection of the human GIMAP6 sequence revealed the presence of a sequence similar to this motif spanning amino acids 3-9 (EEEYEQI) of the protein. Surprisingly, however, when we mutated either of the two key residues in this sequence to alanine (Y6 to A or I9 to A), there was no effect on the ability of the protein to bind to GABARAPL2 (Figure 3A). Moreover, consistent with this result, removal of amino acids 1-36 (i.e. most of the sequence upstream of the GTPase/AIG1 domain of the protein (amino acids 41-241)) also had no effect (Figure 3B). In marked contrast, removal of the C-terminal domain of GIMAP6 (amino acids 244-292), which largely corresponds to a C-terminal α-helical extension of GIMAP6 downstream of the AIG1 domain, completely abolished its ability to bind to GABARAPL2. Indeed we found that removal of the 10 terminal amino acid residues (amino acids 283-292) was sufficient to prevent the interaction (Figure 3B). Interestingly, these final 10 amino acids have no obvious sequence homology to that of an AIM sequence. 

Inspection of an alignment of GIMAP6 orthologues from various mammalian species (Figure S5) shows that, whereas there is very high conservation within the GTPase/AIG1 domain (outlined in black), outside of that region more divergence is apparent. Particularly noticeable is that three rodent species (mouse, rat and chinese hamster), but not the mole rat, have truncated C-terminal domains compared with several other mammalian species, including human. Consistent with our observation that it is the C-terminal region of GIMAP6 which is required for interaction, we were able to demonstrate only a very weak interaction between mouse GIMAP6 and GABARAPL2 (Figure S6). 

Although these results showed a requirement for the C-terminal region of the protein, when we introduced mutations into the GTPase domain of GIMAP6, we could show that this region too was important. Introduction of mutations into the probable G1 (G50D and S54N), G2 (T76A), or G3 GTPase subdomains (D95A and N98A) prevented GIMAP6-GABARAPL2 interaction (Figure 3C), indicating that the structure and possibly the nucleotide binding capacity of the GTPase domain is important for its interaction with GABARAPL2. However, mutation of the putative G4 domain (D167A) had no effect on binding. We also mutated arginine-134 to alanine, as work on the structure of GIMAP2 has indicated that the corresponding residue (arginine-117) is involved in homodimerization [2], but this change did not affect the GIMAP6-GABARAPL2 interaction. 

We then investigated the regions of GABARAPL2 required for its interaction with GIMAP6. Deletion of the N-terminal 10 amino acids of GABARAPL2 was sufficient to prevent the interaction (Figure 3D). GABARAPL2 shares with other members of the Atg8 family a highly conserved glycine residue (G116) close to its C-terminus, at which proteolysis followed by lipidation can occur. This allows the protein to become membrane-anchored to autophagosomes [25]. In contrast to the removal of the first 10 amino acids of the protein, mutation of this residue to alanine, which prevents the occurrence of proteolysis/lipidation, did not prevent the interaction of GABARAPL2 with GIMAP6 (Figure 3D), suggesting that the interaction can occur in the cytosol before GABARAPL2 becomes membrane-associated.

### GIMAP6 regulates the cellular GABARAPL2 level

**Figure 4:**
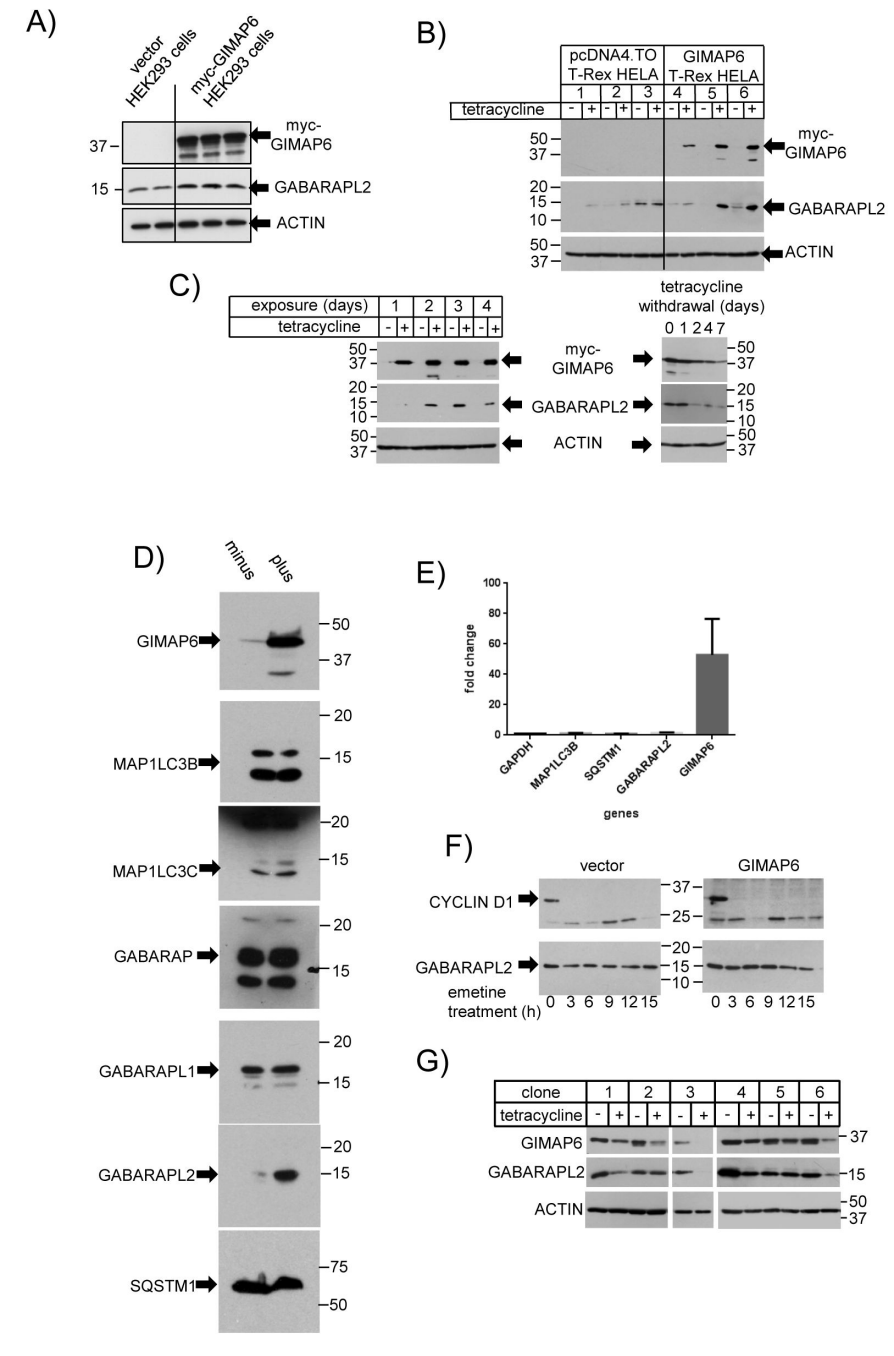
GIMAP6 over-expression leads to the induction of GABARAPL2. A) Cells lysates were prepared from three myc-GIMAP6 HEK293 cell lines (lanes 3-5) or two cell lines carrying the corresponding vector (lanes 1-2) and expression of GIMAP6, GABARAPL2, and β-actin analysed by Western blotting as indicated. B) Three T-Rex^TM^-HeLa cell lines carrying plasmid pcDNA4.TO (lanes 1-3) or three myc-GIMAP6 T-Rex HeLa cell lines (lanes 4-6) were grown in the presence or absence of 2 µg/ml tetracycline for four days. Cells lysates were prepared and analysed for myc-GIMAP6, GABARAPL2 or β-actin expression, as indicated, by Western blotting. C) A myc-GIMAP6 T-Rex Hela cell line was grown for various times in the presence or absence of tetracycline. After 4 days, some dishes of cells that had been grown in the presence of tetracycline were extensively washed and maintained in the absence of tetracycline for further time intervals. At each time-point, cell lysates were prepared and analysed for GIMAP6, GABARAPL2 or β-actin expression (with primary antibodies: rat anti-GIMAP6 monoclonal antibody MAC 445, rat anti-GABARAPL2 MAC446, and mouse anti-β-actin monoclonal antibody AC-15 respectively, followed by the corresponding HRP-conjugated second antibodies) by Western blotting. The experiment was performed on both clones 5 and 6 from panel B with similar results – that from clone 6 is shown. D) A myc-GIMAP6 T-Rex HeLa cell line was incubated in the presence (plus) or absence (minus) of tetracycline for four days. Lysates were prepared, separated by SDS-PAGE, and Western blotted for the expression of GIMAP6 (using rat monoclonal antibody, MAC445), GABARAPL2 (rat monoclonal antibody, MAC446), or other Atg8 members and SQSTM1, using antibodies as detailed in the Materials and Methods section. The results shown are representative of two independent experiments. E) Total RNA was isolated from a myc-GIMAP6 T-Rex HeLa cell line which had been grown in the presence or absence of tetracycline for four days. qPCR was performed as described in the Materials and Methods section. Expression levels were normalised between samples to GAPDH and then the levels of individual RNA species represented as a fold-stimulation of the plus-tetracycline samples relative to those from cells maintained in the absence of tetracycline. Data are presented as mean ± range of two independent experiments. F) myc-GIMAP6 HEK293 cells (right-hand panels) or the corresponding vector cells (left-hand panels) were treated with 10 µM emetine for the indicated times. Cell lysates were prepared and analysed by SDS-PAGE and Western blotting using either anti-GABARAPL2 rat monoclonal MAC446 or anti-β-ACTIN followed by the appropriate HRP-conjugated secondary antibodies. The result shown is representative of two independent experiments. G) Individual clones of TRex-Jurkat cells carrying GIMAP6 shRNA sequences were either treated for 4 days with 1 µg/ml tetracycline or were similarly maintained but in the absence of tetracycline. Cell lysates were prepared and assayed for GIMAP6 (using mAb MAC445) GABARAPL2 (using mAb MAC446) or β-ACTIN expression. Clones 1-3 carried shRNA1 and clones 4-6, shRNA2 (see Materials and Methods). The gap between clones 2 and 3 reflects the removal of intermediate lanes because of inconsistent cell recovery in those samples.

In the course of our studies, we noticed that GIMAP6 over-expression might be affecting intracellular levels of GABARAPL2. For example, HEK293 cells constitutively engineered to express exogenous myc-tagged human GIMAP6 (termed myc-GIMAP6 HEK293), also expressed higher levels of endogenous GABARAPL2 when compared with cells carrying vector plasmid alone (Figure 4A). To address this potential effect directly, we introduced a myc-tagged human GIMAP6 under the control of a tetracycline-regulated promoter in plasmid vector pcDN4.TO into the T-Rex^TM^-HeLa cell line (Invitrogen) – termed the myc-GIMAP6 T-Rex HeLa cell line. Cells carrying the pcDNA4.TO vector alone expressed no detectable human GIMAP6 and very low levels of GABARAPL2, with or without tetracycline treatment (Figure 4B). However, cells carrying the GIMAP6 construct showed strong induction of GIMAP6 expression in response to tetracycline treatment with only very low levels of expression being detectable in the absence of the inducer. In addition, these cells showed degrees of increased GABARAPL2 expression which varied from small (cell line 4) to substantial (cell lines 5 and 6) following tetracycline treatment (Figure 4B). The increase appeared to reflect the relative levels of GIMAP6 expression in the different cell lines and would suggest that GIMAP6 is capable of directly regulating the intracellular levels of GABARAPL2: interestingly, when we performed a time-course of induction of GIMAP6 by tetracycline, we observed that the induction of GABARAPL2 occurred more slowly than that of GIMAP6 (Figure 4C, left hand panel), consistent with the idea that GIMAP6 is capable of modulating GABARAPL2 levels. Withdrawal of tetracycline from the cell line resulted in a reduction in the levels of GIMAP6, returning to close to basal levels in approximately seven days (Figure 4C right hand panel), and the level of GABARAPL2 detected fell in parallel to that of GIMAP6. In contrast to the increase in GABARAPL2 seen following tetracycline-induced GIMAP6 up-regulation, Western blot analysis of the levels of other Atg8 family members in the cells (as before, MAP1LC3A was not detectable) indicated that none of the other family members were increased (Figure 4D), again supporting the idea of a specific relationship between GIMAP6 and GABARAPL2 compared with other Atg8 family members. We also assayed the effect of the tetracycline-mediated GIMAP6 induction on the levels of another protein of key importance in autophagic mechanisms, SQSTM1 (otherwise known as p62), which was also unchanged (Figure 4D).

We were interested to try to determine how GIMAP6 regulated GABARAPL2 levels. The first possibility we considered was that GIMAP6 might be affecting the levels of *GABARAPL2* mRNA. However, comparison by qPCR of mRNA levels following four days of tetracycline treatment with untreated controls, indicated that whilst *GIMAP6* mRNA levels were increased approximately 50-fold, those of *GABARAPL2* averaged 1.4-fold (Figure 4E). For comparison *MAP1LC3B* mRNA was increased 1.2-fold and *SQSTM1*, reduced to 0.9-fold (Figure 4E), changes which produced no detectable changes in the levels of the corresponding proteins (see Figure 4D). This suggests that the increase in GABARAPL2 protein levels is unlikely to be due to increased levels of the corresponding mRNA (but see discussion). 

A second possibility to consider is that GABARAPL2 is stabilised by formation of a complex with GIMAP6, thereby increasing its half-life. As our antisera raised to GABARAPL2 failed to immunoprecipitate GABARAPL2, we could not perform classical pulse-chase studies to determine the half-life of GABARAPL2 in cell lines in the absence or presence of GIMAP6. Therefore, cells were treated with emetine to block protein synthesis and the subsequent disappearance of GABARAPL2 assessed by Western blotting. As, in the absence of GIMAP6 induction, the levels of GABARAPL2 were very low in the myc-GIMAP6 T-Rex HeLa cell lines, assessment of GABARAPL2 disappearance in those cells proved technically difficult. Therefore the study was performed on stably-transfected HEK293 cells engineered to over-express myc-GIMAP6 (myc-GIMAP6 HEK293 cells) or carrying the corresponding vector. These cells inherently express higher levels of GABARAPL2 than the myc-GIMAP6 T-Rex HeLa cell lines even in the absence of GIMAP6. However, after treatment with emetine for 15 h, no reduction in GABARAPL2 levels was detected even in the absence of GIMAP6 (Figure 4F). Treatment for longer periods resulted in increased cell rounding and detachment from the surface of the dish, suggestive of cell death, thus preventing longer time-course analysis. Similar results were obtained in cells over-expressing GIMAP6. As a positive control for the effect of emetine, parallel Western blots were probed with an antibody to a protein known to have a short half-life, CYCLIN D1 [29]. As expected, this protein was undetectable after only 3h of emetine treatment (Figure 4F). These results indicate that, even in the absence of GIMAP6, GABARAPL2 has an inherently long half-life in these cells. However we do not dismiss the possibility that the half-life is increased further in the presence of GIMAP6. 

To address the issue of whether endogenously-expressed GIMAP6 modulates GABARAPL2 levels, we established two sets of cell clones carrying different shRNA sequences targeted at the *GIMAP6* RNA sequence under the control of a tetracycline-inducible promoter in the mammalian T-REx^TM^ Jurkat cell line (Life Technologies). Of the clones generated, many showed a reduction in the endogenous levels of GIMAP6 protein after treatment with tetracycline for four days (Figure 4G). In the majority of those, a reduction in the levels of GABARAPL2 protein was also observed (e.g. clones 1, 3, 4, and 6 in Figure 4G), indicating that changes in the levels of endogenous GIMAP6 protein can affect GABARAPL2 levels. However, in some clones (e.g. 2 and 5 in Figure 4G), whilst a reduction in GIMAP6 was observed after shRNA induction, no reduction in GABARAPL2 levels were observed. We have no explanation for this phenomenon, but it may suggest that multiple factors in a cell can regulate GABARAPL2 levels, of which GIMAP6 is only one. 

### Response of GIMAP6 to cell starvation and mTOR kinase inhibition

**Figure 5:**
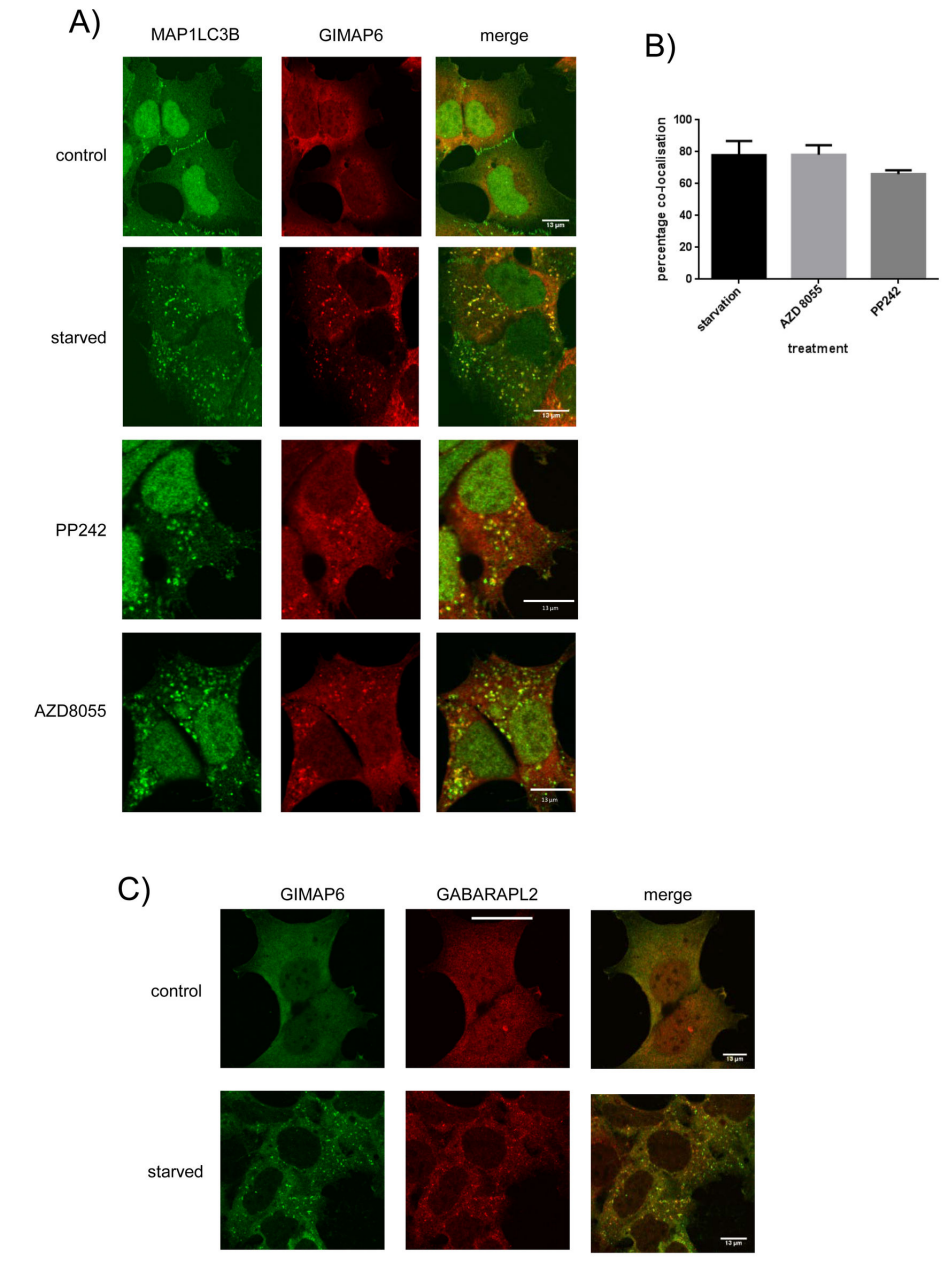
Over-expressed GIMAP6 is recruited to MAP1LC3B positive autophagosomes on induction of autophagy. A) myc-GIMAP6 HEK293 cells were grown on coverslips and were either left untreated (control) or maintained in starvation medium (starved) or treated with mTOR inhibitors AZD8055 (1 µM final concentration) or PP242 (0.4 µM) for 90 min. Cells were then processed for immunocytochemistry using rat anti-human GIMAP6 monoclonal antibody MAC445 or rabbit anti-MAP1LC3B (Sigma product number L7543) followed by an Alexafluor 568-conjugated anti-rat IgG or an Alexafluor 488-conjugated anti-rabbit IgG, respectively. Images were captured using an Olympus FV1000 imaging system. B) Graphical representation of the co-localisation of GIMAP6 puncta with MAP1LC3B puncta, and are shown as a mean percentage ± SD for each autophagic stimulus. Analysis was performed using Imaris software. 7-8 fields of approximately 10 cells/field were viewed for each analysis. C) Cells were grown and treated as in A). They were then stained for GIMAP6 using a rabbit anti-human GIMAP6 polyclonal antiserum and for GABARAPL2 using rat anti-GABARAPL2 mAb MAC446 followed by an Alexafluor 488 conjugated goat anti-rabbit IgG and an Alexafluor 568-conjugated goat anti-rat IgG, respectively. Images were captured using an Olympus FV1000 imaging system. In panels A and C the scale bar represents 13 µm. The results shown in panels A and C are representative of three independent experiments.

Members of the Atg8 family, including GABARAPL2, are recruited to autophagosomes on induction of autophagy (see 25 for review). Because of the association of GIMAP6 with GABARAPL2, we investigated the effect of autophagic induction on the intracellular localization of GIMAP6. Myc-GIMAP6 HEK293 cells, were starved for 90 minutes and the distribution of myc-GIMAP6 compared with that of the classic autophagosomal marker MAP1LC3B. Before starvation, the myc-tagged protein was seen to be predominantly cytosolic (Figure 5A), consistent with the apparent absence of a hydrophobic transmembrane-anchoring sequence in the protein. By contrast, MAP1LC3B was distributed in both cytosol and nucleus in control cells (the latter predominantly), a result consistent with previous reports on the distribution of both EGFP-MAP1LC3B and MAP1LC3B [30]. Under starvation conditions, however, GIMAP6 re-located to punctate structures which were also labelled by the anti-MAP1LC3B antibody, indicating that GIMAP6 was relocating to autophagosomes (Figure 5A). A similar starvation-induced localization of GIMAP6 was observed using an anti-human GIMAP6 monoclonal antibody instead of the anti-myc antibody (Figure S7). To further check that the starvation–induced puncta were related to the induction of autophagy, we treated the cell line with either of two TOR kinase inhibitors, AZD8055 and PP242, which induce autophagy downstream of the inhibition of mTOR. Treatment with either inhibitor resulted in the formation of GIMAP6- and MAP1LC3B-positive puncta, suggesting strongly that the puncta observed on starvation were caused by the induction of autophagy (Figure 5A). Analysis suggested that 70-80% of the GIMAP6 puncta co-localised with MAP1LC3B puncta following autophagic stimulus by any of the treatments (Figure 5B). Importantly, GIMAP6 also co-localised with GABARAPL2-immunoreactive punctate structures on starvation (Figure 5C) at a coincidence of 55.5 ± 8.9 (1 SD) %, indicating that the GIMAP6-GABARAPL2 complex we have observed may be responsible for GIMAP6 recruitment to autophagosomes. 

**Figure 6:**
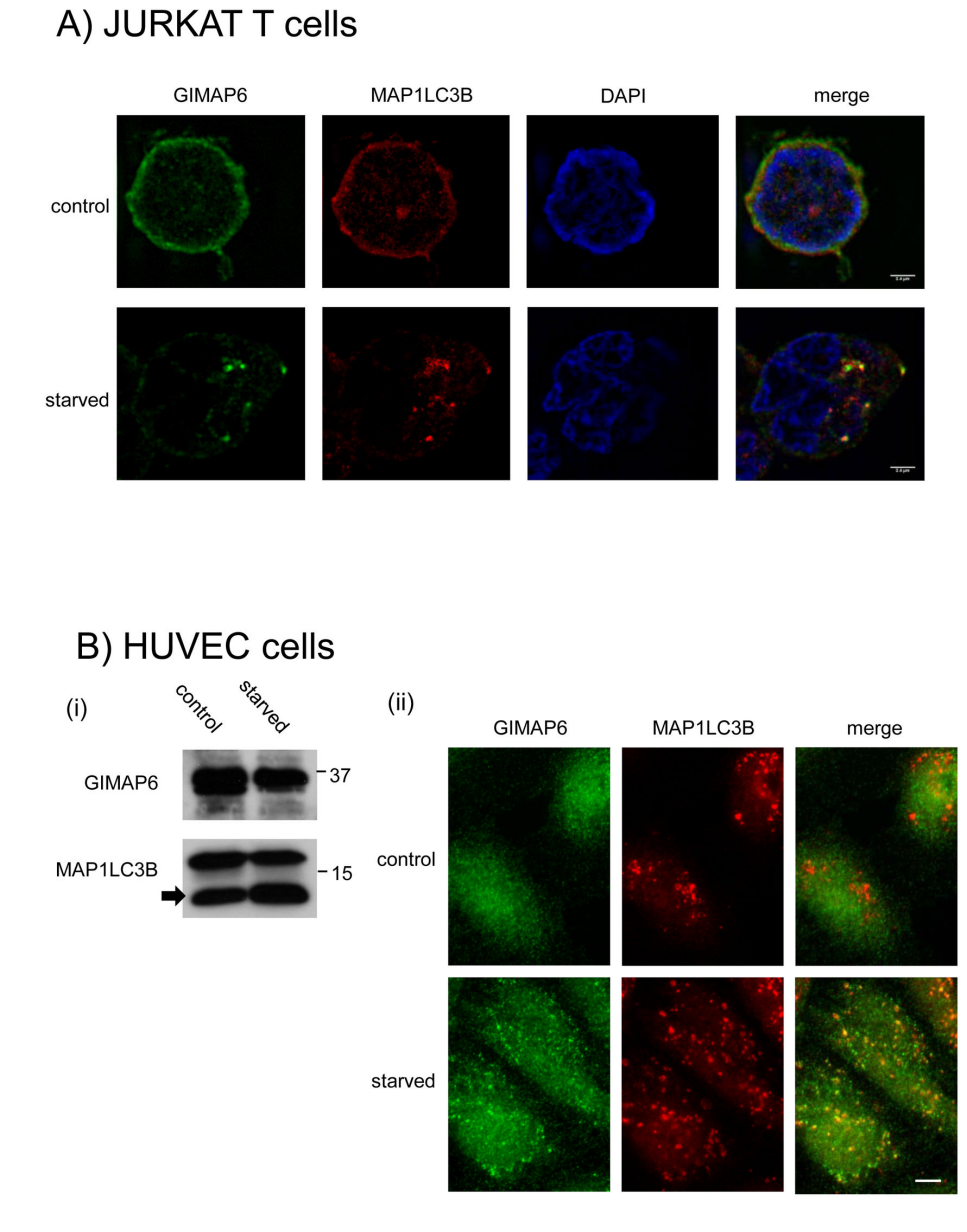
Endogenously expressed GIMAP6 is relocated to punctate structures in response to starvation A) Jurkat-T cells were grown on poly-L-lysine-treated coverslips and were either left untreated or maintained in starvation medium for 90 min. Cells were then processed for immunocytochemistry using rat anti-human GIMAP6 monoclonal antibody MAC445 or rabbit anti-MAP1LC3B (Sigma product number L7543) followed by an Alexafluor 488-conjugated anti-rat IgG or an Alexafluor 568-conjugated anti-rabbit IgG, respectively. Note that on starvation cells flatten slightly on to the coverslips and thus have a slightly different appearance with respect to the nucleus. Images were captured using a Nikon N-SIM super-resolution system with a CFI Apo TIRF 100x oil objective lens (N.A. 1.49). Scale bar represents 2.4 µm. B) (i) Cell lysates prepared from control or HUVEC cells starved as described in Figure 6A were analysed by Western blotting using rat mAb MAC445 to GIMAP6 or a rabbit polyclonal antibody to MAP1LC3B as indicated. MAP1LC3B-II is indicated by an arrow. (ii) HUVEC cells were grown and then were either starved or left untreated and subsequently processed for immunocytochemistry as described in Figure 6A. Fluorescence microscopy was performed using an Axio Imager.D2 microscope (Carl Zeiss Microscopy) with a 100 x oil emersion objective. The results shown in each panel are representative of three independent experiments.

As GIMAP6 was over-expressed in these cells, we were concerned that the re-location observed on starvation could be artifactual. However, as shown above, GIMAP6 is expressed endogenously in Jurkat-T cells (see Figure 1E), and we therefore attempted to visualise the endogenous protein in these cells. Because of the small amounts of cytoplasm surrounding the nucleus in these cells, imaging is technically demanding. Nevertheless, high resolution fluorescence microscopy of the cells indicated that both GIMAP6 and LC3 are normally present spread diffusely throughout the cell (Figure 6A). However, on starvation, both GIMAP6 and LC3 become re-distributed to punctate structures, with some clear co-localisation (Figure 6A). This strongly suggests that the results observed in the myc-GIMAP6 HEK293 cells reflect the normal response of GIMAP6 to starvation. 

Because of the difficulty of imaging Jurkat T cells, we sought another cell line that expressed GIMAP6 endogenously. It has recently been reported that GIMAP6 is expressed by endothelial cells [31], and, consistent with this, we were able to demonstrate GIMAP6 expression in primary human vascular endothelial cells (HUVEC) - Figure 6B(i). Starvation of these cells resulted in increased levels of MAP1LC3B-II (arrowed in Figure 6B(i)), indicative of the induction of autophagy. Immunocytochemical analysis of the distribution of GIMAP6 in the HUVEC cells showed a cytoplasmic localisation in the control cells (Figure 6B(ii)). On starvation, discrete punctate structures were observed, which co-localised with MAP1LC3B, indicating that, even in primary cells, GIMAP6 can be recruited to autophagosomes.

As GABARAPL2 has been described to be essential for autophagy [32], we determined whether GIMAP6 over-expression had any measurable effect on autophagy as assessed by the formation of MAP1LC3B-II from MAP1LC3B-1 (see 27). The production of MAP1LC3B-II in response to cell starvation in the presence or absence of chloroquine (to inhibit proteolytic intra-lysosomal cleavage) was assessed in all of the over-expressing cell lines described in this paper, i.e.: the Biot-GIMAP6-His myc-BirA-Jurkat cell line and the corresponding vector control; the myc-GIMAP6 T-Rex HeLa cell line with or without tetracycline treatment to induce GIMAP expression; a myc-GIMAP6 HEK293 cell line and parental vector cells. Typical results are shown in Figure S8. In none of the three groups of cell lines were any clear differences seen in the ratio of MAP1LC3B-II/ACTIN levels between cells over-expressing GIMAP6 and the corresponding basal cells, either in untreated cells or following starvation and/or chloroquine treatment. To investigate the effect of GIMAP6 in more detail, the experiment with the Biot-GIMAP6-His myc-BirA-Jurkat cell line and the corresponding vector control cells was performed three times. These data are shown in Figure S8 panel D. No statistically significant differences were found with any treatment between vector cells and cells over-expressing GIMAP6. In addition when the effect of GIMAP6 over-expression in myc-GIMAP6 HEK293 cells on the number of LC3 or SQSTM1 puncta (spots) formed in response to starvation was studied, no statistically significant difference was observed between the control (vector) cells and cells over-expressing GIMAP6 (Figure S9). We deduce from these data that GIMAP6 expression does not have a simple relationship to the bulk process of autophagy, as defined by MAP1LC3B-I to MAP1LC3B-II conversion, despite the interaction of the protein with GABARAPL2.

**Figure 7:**
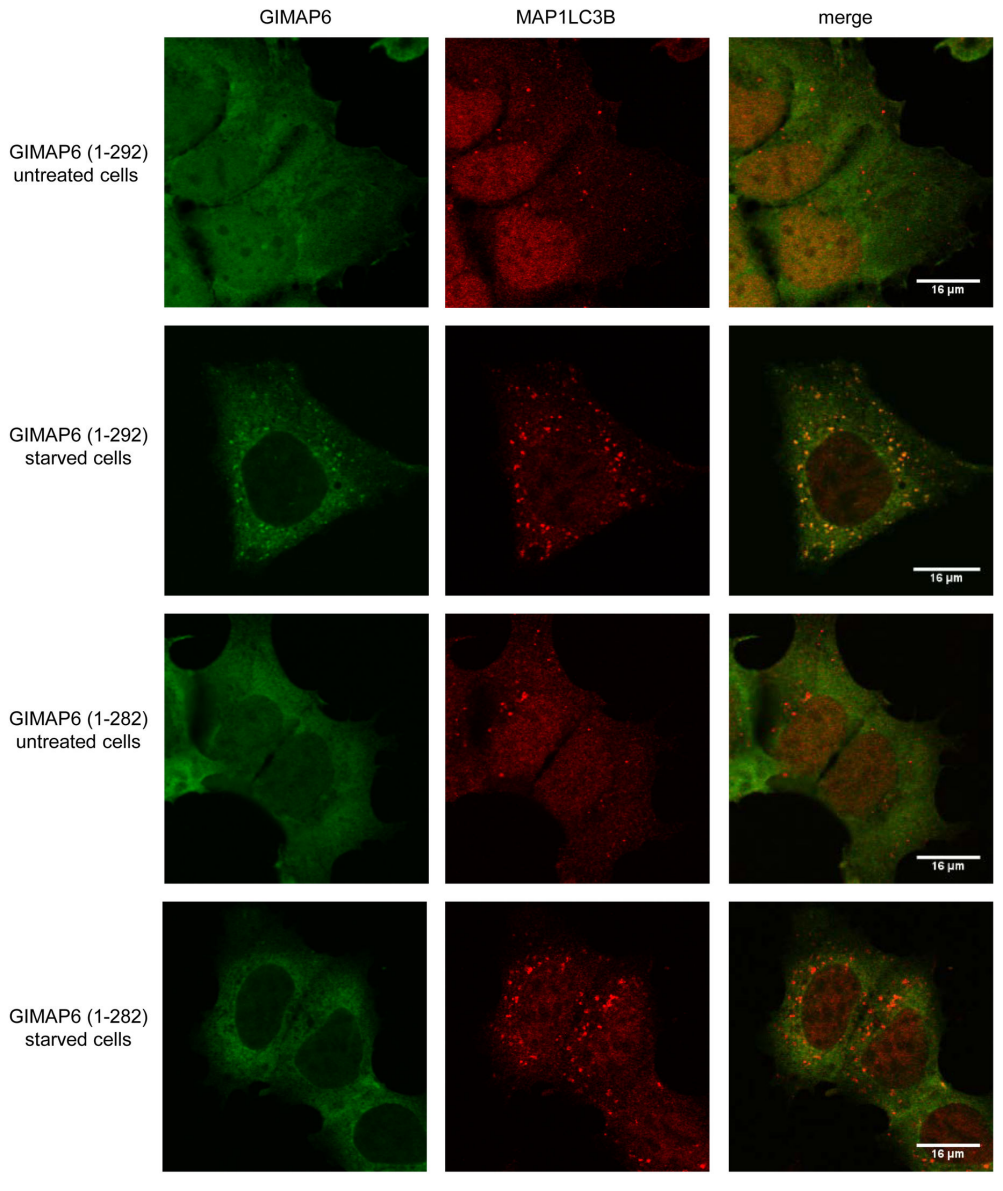
The C-terminal 10 amino acids of GIMAP6 are required for its recruitment to autophagosomes. A stable HEK293 cell line expressing a myc-tagged GIMAP6 lacking the C-terminal 10 amino acids and the myc-GIMAP6 HEK293 cell line were either left untreated or were treated with starvation medium for 90 min. Cells were then processed for immunocytochemistry using rat anti-human GIMAP6 monoclonal antibody MAC445 or rabbit anti-MAP1LC3B (Sigma product number L7543) followed by an Alexafluor 488-conjugated anti-rat IgG or an Alexafluor 568-conjugated anti-rabbit IgG, respectively. Images were captured using an Olympus FV1000 imaging system. GIMAP6 (1-292) indicates the full-length protein and GIMAP6 (1-282) the truncated form. Scale bar represents 16 µm. The results shown are representative of three independent experiments.

We therefore asked whether the interaction of GIMAP6 with GABARAPL2 is required for the recruitment of GIMAP6 to autophagosomes, to perhaps either permit its selective degradation or to facilitate a specific function at the vesicles. Stable HEK293 cells were generated which expressed a truncated form of GIMAP6 (amino acids 1-282) which we have shown fails to interact with GABARAPL2 (see Figure 3). When autophagy was induced in these cells by starvation, in contrast to full length GIMAP6 (GIMAP6 (amino-acids 1-292)) no recruitment of the truncated protein to autophagosomes was observed, although MAP1LC3B formed puncta normally (Figure 7). Whilst we cannot dismiss the possibility that amino acids 283-292 of GIMAP6 interact with other proteins that mediate its recruitment to autophagosomes, it seems likely that it is the disruption of the GIMAP6-GABARAPL2 interaction which prevents the recruitment.

**Figure 8:**
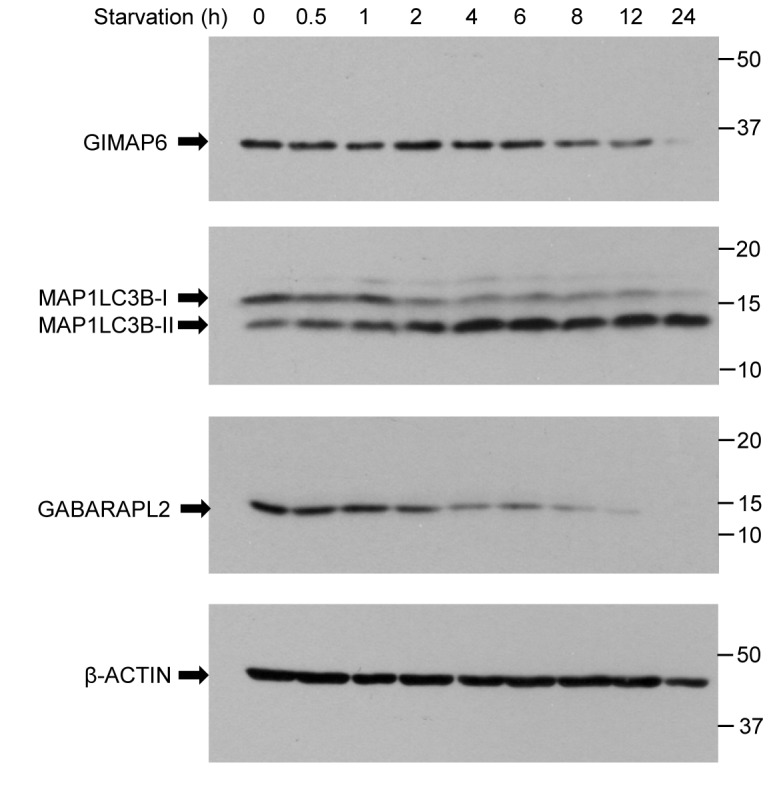
GIMAP6 is degraded in cells during starvation-induced autophagy. Jurkat T cells were maintained in starvation medium for the indicated times. Lysates were prepared, separated by SDS-PAGE, and Western blotted for the expression of GIMAP6 (using rat monoclonal antibody, MAC445), GABARAPL2 (rat monoclonal antibody, MAC446), and MAP1LC3B and β-ACTIN as detailed in the Materials and Methods section. The result shown is representative of two independent experiments.

As GIMAP6 is recruited to autophagosomes on starvation, it was of interest to determine whether this recruitment might be associated with GIMAP6 turn-over. We therefore maintained Jurkat T cells in starvation medium for varying lengths of time, and then analysed lysates for levels of GIMAP6, GABARAPL2, MAP1LC3B and β-ACTIN. As expected, starvation stimulated conversion of MAP1LC3B-I to MAP1LC3B-II, indicative of autophagy (Figure 8). The MAP1LC3B-II produced reaches a steady state which is maintained to at least 12 h. In contrast GABARAPL2 levels fall rapidly on starvation and are almost undetectable by 12 h. GIMAP6 disappears with a time-course intermediate between that of MAP1LC3B and GABARAPL2, with a clear reduction by 12 h. This suggests that at least part of the function of the recruitment of GIMAP6 to autophagosomes is to selectively mediate its destruction.

## Discussion

An unbiased biochemical search for proteins interacting with GIMAP GTPases led us to identify GABARAPL2 as a major binding partner for GIMAP6. GABARAPL2 is a homologue of yeast Atg8, a ubiquitin-like protein involved in cargo recruitment into autophagosomes and in their biogenesis. The best-studied of the mammalian Atg8 homologues is MAP1LC3B and the conversion between its unmodified form (MAP1LC3-I) and its phosphatidylethanolamine-modified form (MAP1LC3-II) constitutes a favoured assay of autophagic activity in cells. 

GABARAPL2 is much less well studied. It was originally reported as a protein involved in intra-Golgi transport [33]. Subsequently, the same laboratory demonstrated that GABARAPL2 (under its alternative name, GATE-16) interacts directly with the N-ethylmaleimide-sensitive factor (NSF) and complexes with the Golgi v-SNARE GOS-28 in an NSF-dependent manner [34], supporting a role for it in intra-Golgi transport. It was reported later that GABARAPL2 could also localise to autophagosomes [35], and is essential for the autophagic process [32]. Interestingly, yeast Atg8, which is essential for autophagy in this species [36,37] will substitute for GABARAPL2 in an *in vitro* mammalian Golgi transport assay [38], supporting the idea that GABARAPL2 (and by extension the other Atg8 homologues) may have multiple roles in mammalian cells. 

The results we present here show that not only do GIMAP6 and GABARAPL2 interact but also both are recruited to autophagosomes on the induction of autophagy. Among other functions (e.g mediating membrane fusion [39]) a major role of members of the Atg8 family is recruitment of proteins to autophagosomes (see (Noda et al, 2010)). These recruited proteins often, but not exclusively, interact with Atg8 family members through an AIM and often multiple members of the Atg8 family will interact with a single AIM-containing protein. For example, NBR1 has been shown to interact with MAP1LC3B, GABARAP and GABARAPL2 [40]. Indeed, in a large–scale analysis, out of 67 Atg8-interacting proteins identified, only 31 were found to interact with single members of the family [41], and GABARAPL2 was found to interact uniquely with only two proteins, UBA5 and WDR62. Furthermore, additional detailed analysis suggested that, in fact, UBA5 may interact with other Atg8 family members (WDR62 was not analysed in more detail). The work we have described here suggests that GIMAP6 has a strong, although not absolute, preference for interaction with GABARAPL2 over other members of the Atg8 family. Furthermore, our cross-linking analysis in Jurkat T cells suggests that substantial proportions of the endogenous pools of both GIMAP6 and GABARAPL2 are associated with each other in the cytosol of these cells under normal conditions. 

 These results suggest an important role for the molecular interaction between GIMAP6 and GABARAPL2. However, the precise significance of this molecular relationship remains unclear. One possibility is that GIMAP6 is a passive cargo that GABARAPL2 escorts for degradation via the autophagolysosomal pathway under particular conditions of stress (e.g. starvation), an idea supported by our observation of GIMAP6 degradation during starvation-induced autophagy (Figure 8). Under such circumstances the close apposition of GABARAPL2 with GIMAP6 in the cytosol could serve to optimise the efficiency of such a clearance of GIMAP6. An alternative possibility is that GIMAP6 is controlling GABARAPL2. We have presented direct evidence that GIMAP6 can modulate the cellular levels of GABARAPL2. Additionally, it is interesting that, despite the identification of an essential interaction sequence at the carboxy terminus of human GIMAP6, the binding between the two proteins appears also to be dependent on the GTPase domain and on key residues within the consensus G motifs that control guanosine nucleotide binding. This suggests that an active GTPase cycle in GIMAP6, by regulating the GABARAPL2-GIMAP6 interaction, could control, for instance, the availability of GABARAPL2 to the autophagy pathway vs. other cellular functions in which it participates. Finally, to draw an analogy with the recent findings on the interaction of the Rab33-GAP, OATL1, with Atg8 family members [42], it is possible that GABARAPL2 could serve as a scaffold from which GIMAP6 may perform (as yet unknown) functions within the autophagic pathway. 

It should be noted that the studies we have reported here suggest that there is no simple role for GIMAP6 in direct modulation of the bulk autophagic pathways. However the findings do indicate a link between the immune system GTPase GIMAP6 and a protein (GABARAPL2) intimately involved in the process of autophagy. This leads one to ask whether engagement in aspects of autophagy or in its regulation might link all of the 7-8 GIMAPs expressed by mammals. Coordinated involvement in a defined intracellular process would be consistent with similarities in the tissue distributions of the GIMAPs (http://biogps.org/?referer=symatlas%20%5C%20goto=genereport&id=121260#goto=welcome), in their expression through T and B cell development [16,43] and in the tight clustering of their genes in the genome. But can what is already known about this GTPase family be accommodated within an autophagy-related framework? The most prominent physiological processes with which the GIMAPs are associated are lymphocyte development and survival, prominently, but not exclusively, in the T cell lineage. Genetic deficiencies in either GIMAP5 or GIMAP1 produce severe peripheral lymphopenias in rodent models. In studies of GIMAP5 this led to the categorisation of this molecule as a pro-survival factor. Furthermore, the demonstration of biochemical and functional interactions of GIMAP5 with members of the Bcl-2 family [1], alongside data apparently locating it to mitochondria [1,44], fitted well with a model in which this and other GIMAPs were proposed to interact with the core apoptotic machinery. However, genetic Bim deficiency, which can restore the T cell compartment in the case of IL-7R deficiency [45] was reported not to prevent lymphopenia caused by the GIMAP5 sphinx mutation [15] and further difficulty for the simple apoptosis model arose from a thorough examination of GIMAP5 expression in mouse, rat and human cells which revealed that it is located not on mitochondria but on lysosomes and multivesicular bodies [24].

We conclude from this that a more indirect relationship with apoptotic processes could be considered for the GIMAPs. In this respect, autophagic mechanisms are an interesting candidate for their primary functional focus: nutrient salvage via catabolic autophagy clearly plays a pro-survival role in cells, but more specialised roles for autophagy, such as the regulation of mitochondrial number and quality by mitophagy (reviewed by Youle and Narendra [46]), themselves may have consequences for lymphocyte survival. Activation of core apoptotic mechanisms is, of course, a likely consequence of autophagic failure or defects, and regulatory links between the two processes have been established. It is interesting to note here that the lymphopenic T cell phenotypes of GIMAP5-deficient rats and mice share features with those of mice in which core autophagy genes (e.g. *Atg*5, *Atg*7) have been ablated conditionally in the T cell lineage. In both cases, intrathymic T cell development is relatively normal while the peripheral T cell population is severely depleted and the remaining T cells [47,48] are unusual in phenotype, with marked absence of resting, naïve T cells [49]. In the ATG5/7-deficient models this developmental hiatus at the thymus-to-periphery transition has been ascribed to a failure of mitophagy. It has been shown that thymocytes normally exhibit a much higher mitochondrial load than peripheral T cells and it is proposed that a programmed and autophagy gene-dependent reduction in this load is necessary to protect T cells from death via oxidative stress in the more oxygen-rich environment of the peripheral compartments. Indeed, measurements of mitochondrial load in thymocytes, recent thymic emigrants and peripheral T cells from GIMAP5-mutant rats have revealed deficient reduction of mitochondrial load in this model (L. Webb, G.W.B., data not shown). Deficiencies in GIMAP5 and Atg7 also impose similar effects deleterious to quiescent haematopoietic stem cells and impair their repopulating capacity [19,50]. Considering other members of the GIMAP family, the phenotype of GIMAP1-deficient T and B cells is more severe than for GIMAP5 [16], but shows a similar developmental catastrophe at the primary-to-secondary lymphoid transitions (thymus- or bone marrow-to-periphery) so a related cellular role can be predicted. For the mouse-specific GIMAP3, a link to a process of mitochondrial segregation observable in heteroplasmic mice has been described [51] and it is plausible that selection by mitophagy might play a role in this phenomenon. More speculatively, a link to autophagy could be suggested for GIMAP2 which is present on the surface of lipid droplets [2]. The latter are known to be subject to regulation by autophagic components in the process termed macrolipophagy [52].

In conclusion we have uncovered a highly specific molecular interaction between a member of the immune-associated GIMAP GTPase family, GIMAP6, and the mammalian Atg8 homologue, GABARAPL2. Further findings demonstrating a re-location of this GTPase to LC3-positive autophagosomes in response to starvation or mTOR inhibition lead us not only to propose a role for this GTPase in autophagy-related processes in lymphocytes but also to speculate more generally on roles in such processes for the other members of the GIMAP family.

## Supporting Information

Figure S1 **Anti-GIMAP6 monoclonal and polyclonal antibodies are selective for GIMAP6 amongst human GIMAP proteins.** Myc-tagged variants of human GIMAP proteins were expressed in HEK293T cells. Lysates were prepared, separated by SDS PAGE and analysed by Western blotting using anti-myc antibody 9E10, or a rat mAb (MAC445) to human GIMAP6 or a rabbit polyclonal antiserum to the same protein.
(TIF)

Figure S2 **Characterisation of the specificity of Atg8 family antibodies.** Lysates prepared from HEK293-T cells transiently transfected with plasmids encoding N-terminally HA-tagged Atg8 family members were analysed by SDS PAGE and Western blotting, using antibodies either as detailed in the Materials and Methods section or with rat anti-human GABARAPL2 monoclonal antibody MAC446 or anti-HA antibody 12CA5 as indicated.
(TIF)

Figure S3 **A 15kDa protein co-purifies with biotinylated GIMAP6 from transiently transfected HEK293Tcells.** HEK293T cells were transiently transfected with a plasmid encoding human GIMAP6 in pcDNA3Biot1His6iresBirA or with the corresponding vector as indicated. Lysates were prepared 48 h later and the biotinylated and associated proteins purified using streptavidin-agarose. The purified proteins were separated by SDS PAGE and the gel silver-stained. The electrophoretic mobilities of the purified GIMAP6 and an associated 15kDa protein are indicated. The result shown is representative of two independent experiments.
(TIF)

Figure S4 **Endogenous GIMAP6 and GABARAPL2 fail to co-immunoprecipitate.** Cell lysates were prepared from Jurkat-T cells which had either been left untreated or starved for 2 h as described in the Materials and Methods section. Lysates were then either analysed directly by SDS-PAGE and Western blotting for expression of GIMAP6 and GABARAPL2 (panel A left hand side) or MAP1LC3B (panel B) or were first immunoprecipitated with rabbit anti human GIMAP6 polyclonal antiserum (I) or the corresponding pre-immune serum (P) prior to SDS PAGE and Western blotting (panel A right hand side).
(TIF)

Figure S5 **Sequence alignment of GIMAP6 protein sequences from various mammalian species.** Protein sequences were either taken directly from the NCBI protein database or were deduced from expressed DNA sequence tags or genomic sequences. The conserved AIG1/GTPase domain is boxed in black and the extended C-terminal regions present in most mammals, but absent from mouse, rat and chinese hamster, boxed in red.
(TIF)

Figure S6 **Mouse GIMAP6 associates weakly with GABARAPL2.** HEK293T cells were transfected with myc-GIMAP6-encoding plasmids with or without GABARAPL2 in pcDNA3Biot1His6iresBirA, as indicated. Lysates were prepared, and biotinylated and associated proteins purified from the lysates using streptavidin-agarose. Western blots of the recovered proteins were probed with HRP-conjugated streptavidin (to show GABARAPL2) or a mouse monoclonal antibody (9E10) to the myc-tag followed by an HRP-conjugated goat anti-mouse IgG (to show GIMAP6). Western blots were developed using Immobilon ECL western blotting substrate. Interpretation of this experiment was complex as mouse GIMAP6 expressed only weakly in our transient assays compared with the human orthologue. However, co-immunoprecipitation of mouse GIMAP6 with GABARAPL2 was demonstrated in two independent experiments.
(TIF)

Figure S7 **The mouse anti-myc mAb 9E10 and a rabbit anti-human GIMAP6 polyclonal antiserum show similar intracellular distribution of myc-GIMAP6.** Myc-GIMAP6 HEK293 cells were either starved for 90 minutes or left untreated and were then processed for immunocytochemistry, using primary antibodies as indicated. The scale bars indicate 16 µm. The results shown are representative of three independent experiments.
(TIF)

Figure S8 **GIMAP6 over-expression does not affect MAP1LC3B-II accumulation.** Cell lines (panel A – Biot-GIMAP6-His myc-BirA-Jurkat cell line or the corresponding parental cell line; panel B – myc GIMAP6 T-Rex HeLa cell line plus or minus tetracycline treatment; panel C – myc-GIMAP6 HEK293 cells or the corresponding vector control cells) were starved for 2 h (panels A and B) or 1.5h (panel C) or left untreated, with or without treatment with chloroquine as indicated. Cell lysates were prepared and analysed by SDS PAGE and Western blotting with antibodies to MAP1LC3B and ACTIN. Resulting X-ray films were scanned and images analysed using ImageJ software to determine MAP1LC3B-II/ACTIN ratios. Panel D – analysis of MAP1LC3B-II/ACTIN ratios for three experiments on the cells from Panel A. Results are shown as mean ± SD (n=3). The presence of chloroquine is indicated by CQ. One-way ANOVA indicated no significant statistical differences between treatments in the absence or presence of GIMAP6.
(TIF)

Figure S9 **GIMAP6 expression does not affect the number of MAP1LC3 or SQSTM1 puncta/cell.** myc-GIMAP6 HEK293 cells and the corresponding vector cells were either starved for 90 min or left untreated and subsequently immunocytochemically stained for MAP1LC3B or SQSTM1 as indicated. Typical images are shown in panel A. The scale bars represent 21 µm. Spots were counted (150-200 cells analysed for MAP1LC3B and 100-150 for SQSTM1 per condition). Analysis was performed using Imaris software. Results are presented as spots/cell ± SD – panel B MAP1LC3B; panel C SQSTM1. No significant difference was seen in the number of spots in the absence or presence of GIMAP6. Immunocytochemical results shown are representative of three independent experiments.
(TIF)
