# Supplementary figures and images for: The Immune System GTPase GIMAP6 Interacts with the Atg8 Homologue GABARAPL2 and Is Recruited to Autophagosomes
Source: PLoS One. 2013 Oct 17;8(10):e77782. doi: 10.1371/journal.pone.0077782 (PMC3804274; doi:10.1371/journal.pone.0077782)

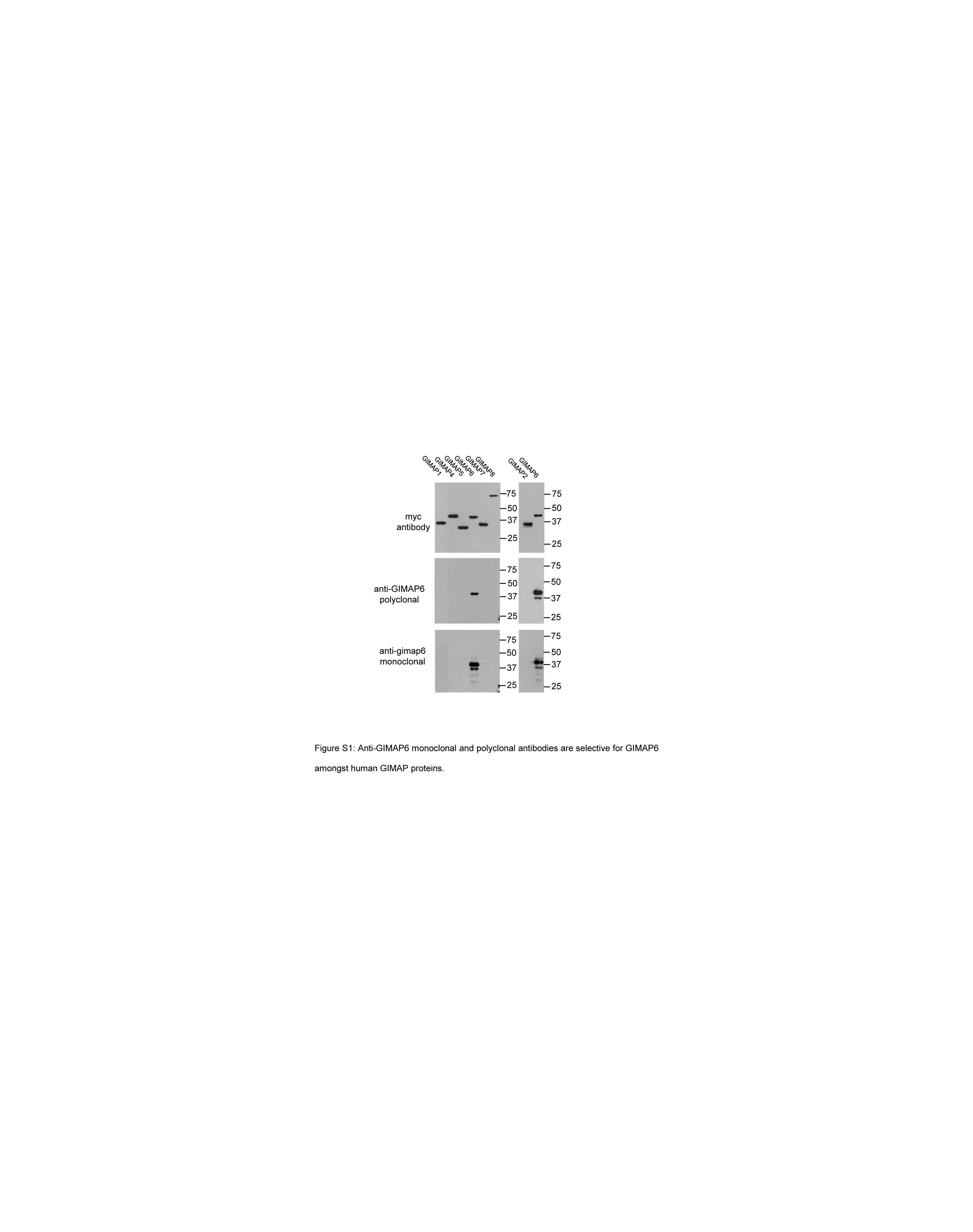

Supplement: Figure S1 — Anti-GIMAP6 monoclonal and polyclonal antibodies are selective for GIMAP6 amongst human GIMAP proteins. Myc-tagged variants of human GIMAP proteins were expressed in HEK293T cells. Lysates were prepared, separated by SDS PAGE and analysed by Western blotting using anti-myc antibody 9E10, or a rat mAb (MAC445) to human GIMAP6 or a rabbit polyclonal antiserum to the same protein. (TIF) [file pone.0077782.s001.tif]

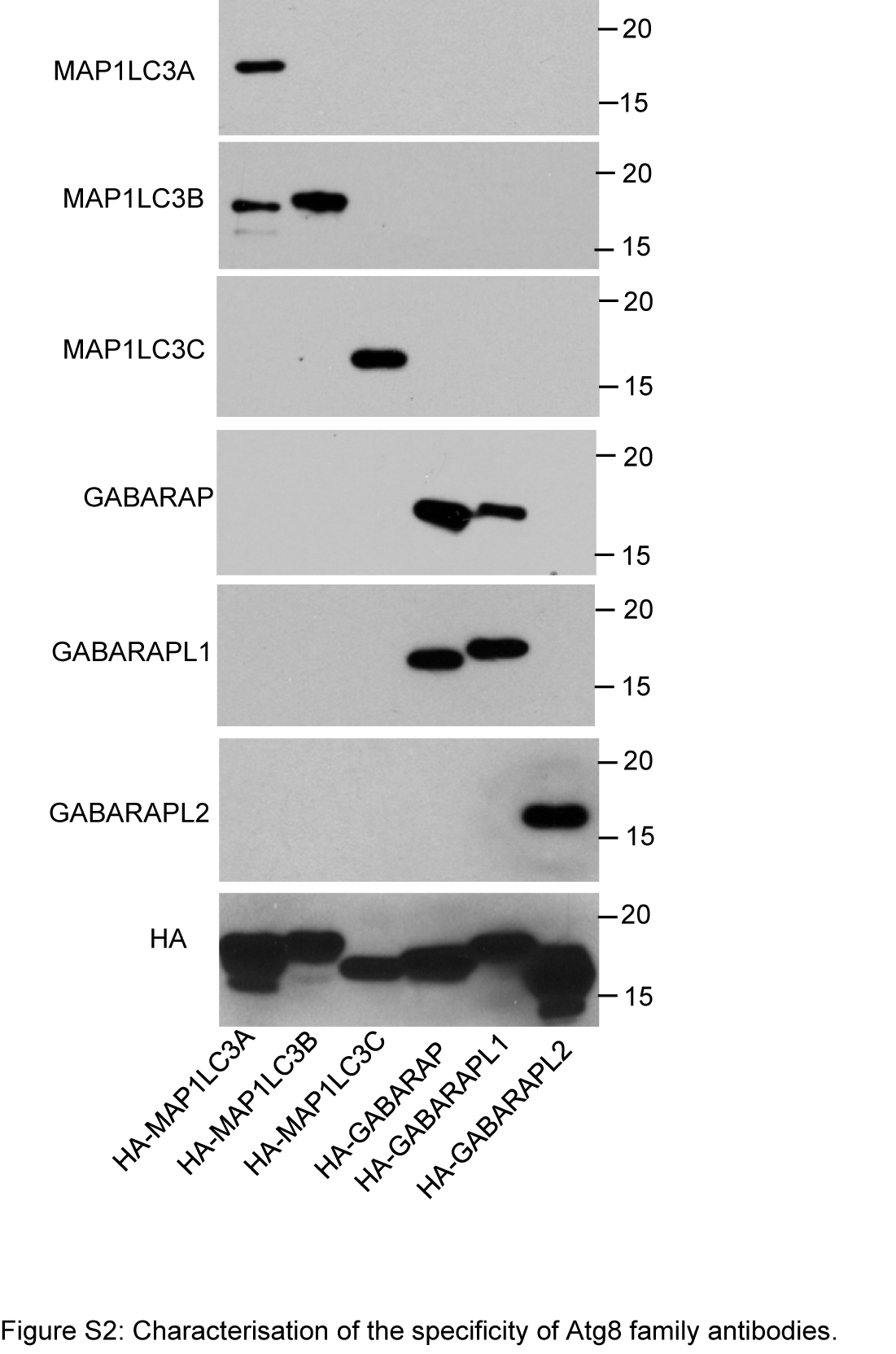

Supplement: Figure S2 — Characterisation of the specificity of Atg8 family antibodies. Lysates prepared from HEK293-T cells transiently transfected with plasmids encoding N-terminally HA-tagged Atg8 family members were analysed by SDS PAGE and Western blotting, using antibodies either as detailed in the Materials and Methods section or with rat anti-human GABARAPL2 monoclonal antibody MAC446 or anti-HA antibody 12CA5 as indicated. (TIF) [file pone.0077782.s002.tif]

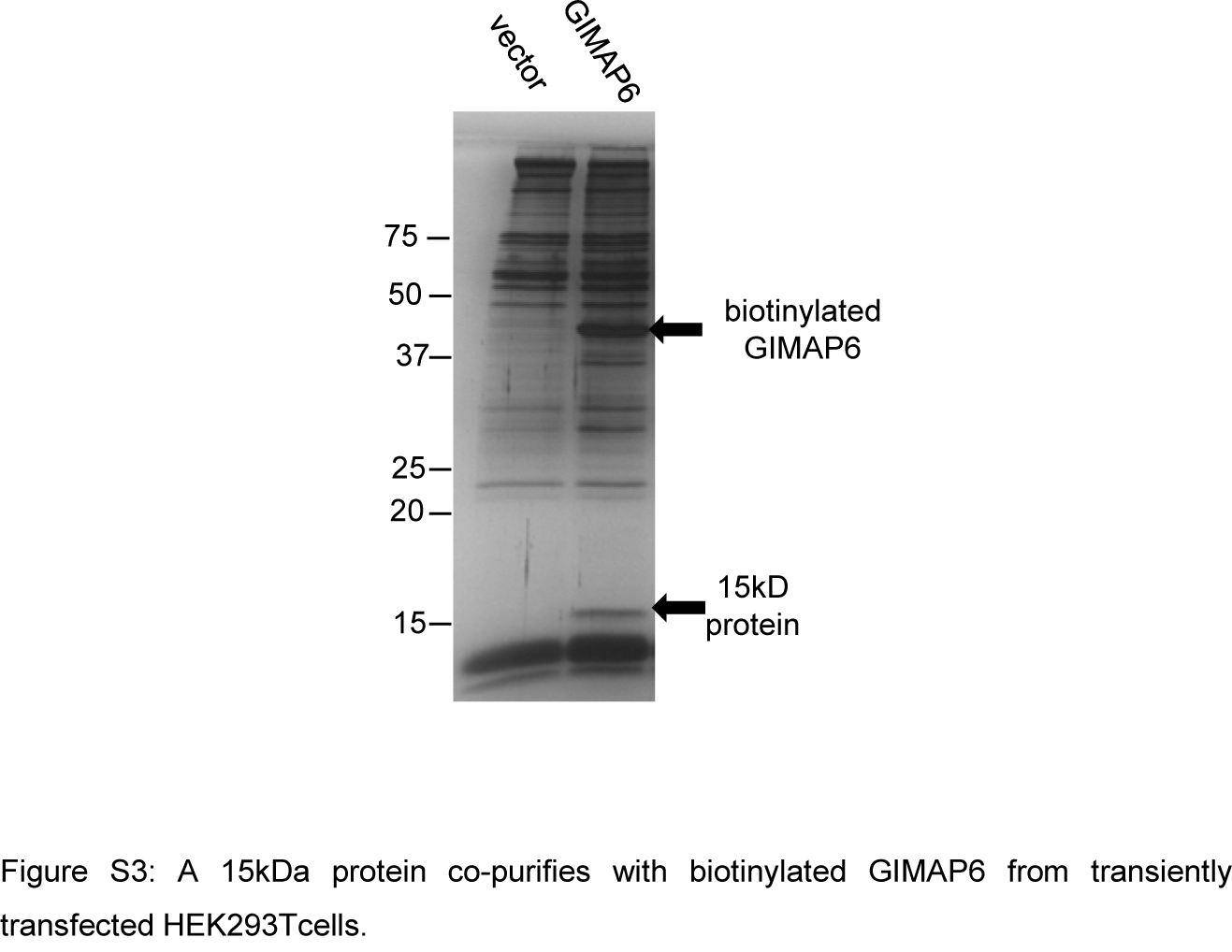

Supplement: Figure S3 — A 15kDa protein co-purifies with biotinylated GIMAP6 from transiently transfected HEK293Tcells. HEK293T cells were transiently transfected with a plasmid encoding human GIMAP6 in pcDNA3Biot1His6iresBirA or with the corresponding vector as indicated. Lysates were prepared 48 h later and the biotinylated and associated proteins purified using streptavidin-agarose. The purified proteins were separated by SDS PAGE and the gel silver-stained. The electrophoretic mobilities of the purified GIMAP6 and an associated 15kDa protein are indicated. The result shown is representative of two independent experiments. (TIF) [file pone.0077782.s003.tif]

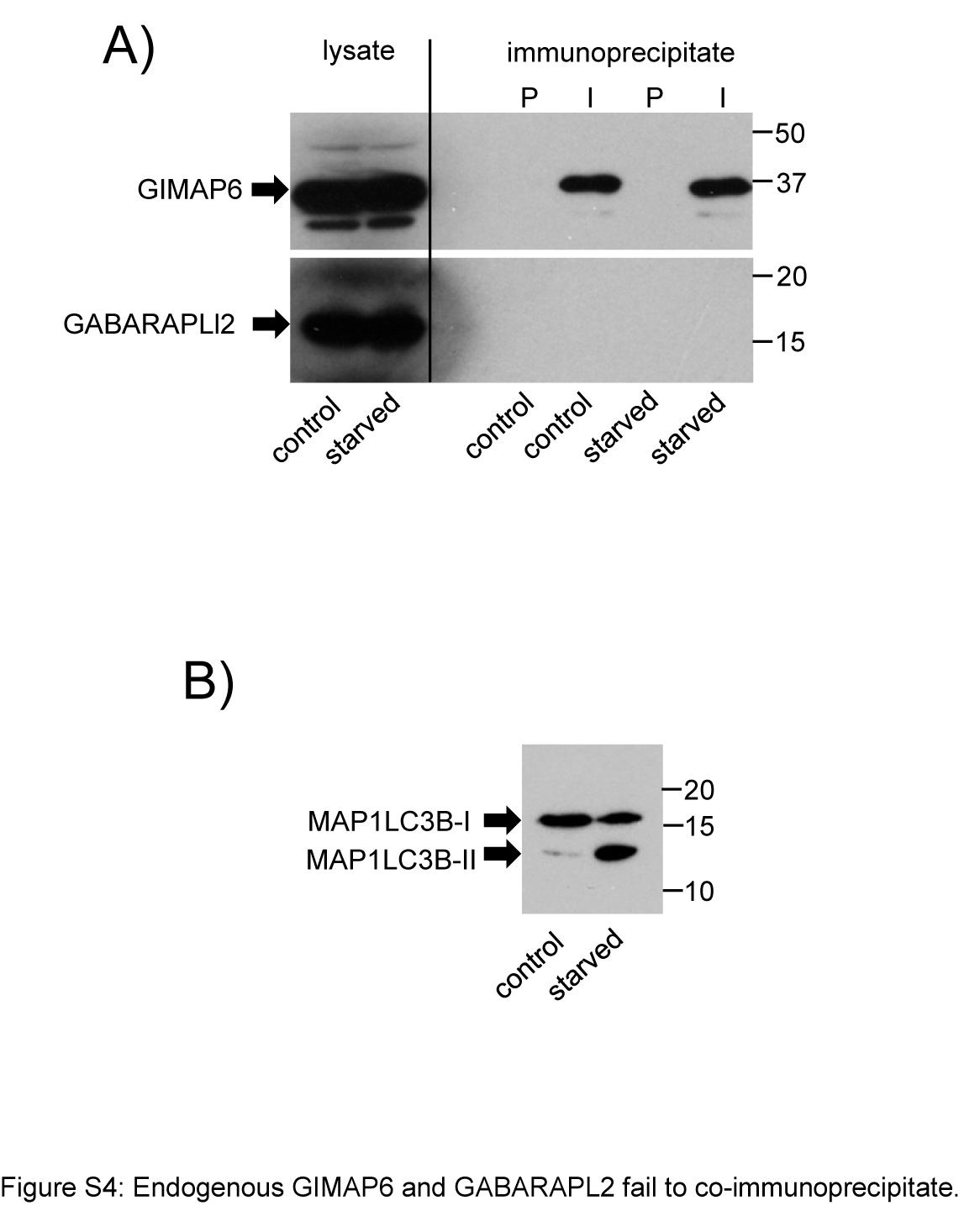

Supplement: Figure S4 — Endogenous GIMAP6 and GABARAPL2 fail to co-immunoprecipitate. Cell lysates were prepared from Jurkat-T cells which had either been left untreated or starved for 2 h as described in the Materials and Methods section. Lysates were then either analysed directly by SDS-PAGE and Western blotting for expression of GIMAP6 and GABARAPL2 (panel A left hand side) or MAP1LC3B (panel B) or were first immunoprecipitated with rabbit anti human GIMAP6 polyclonal antiserum (I) or the corresponding pre-immune serum (P) prior to SDS PAGE and Western blotting (panel A right hand side). (TIF) [file pone.0077782.s004.tif]

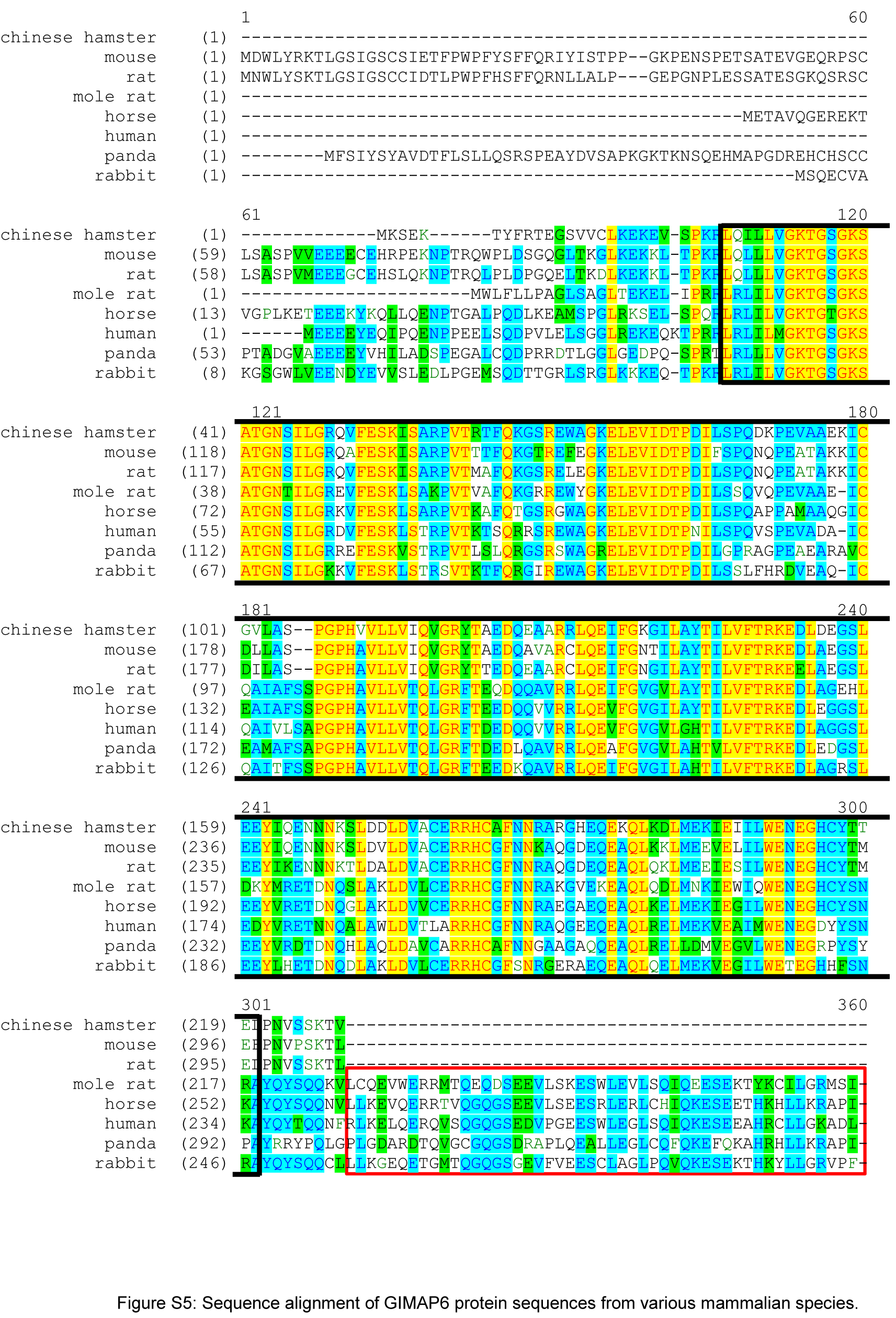

Supplement: Figure S5 — Sequence alignment of GIMAP6 protein sequences from various mammalian species. Protein sequences were either taken directly from the NCBI protein database or were deduced from expressed DNA sequence tags or genomic sequences. The conserved AIG1/GTPase domain is boxed in black and the extended C-terminal regions present in most mammals, but absent from mouse, rat and chinese hamster, boxed in red. (TIF) [file pone.0077782.s005.tif]

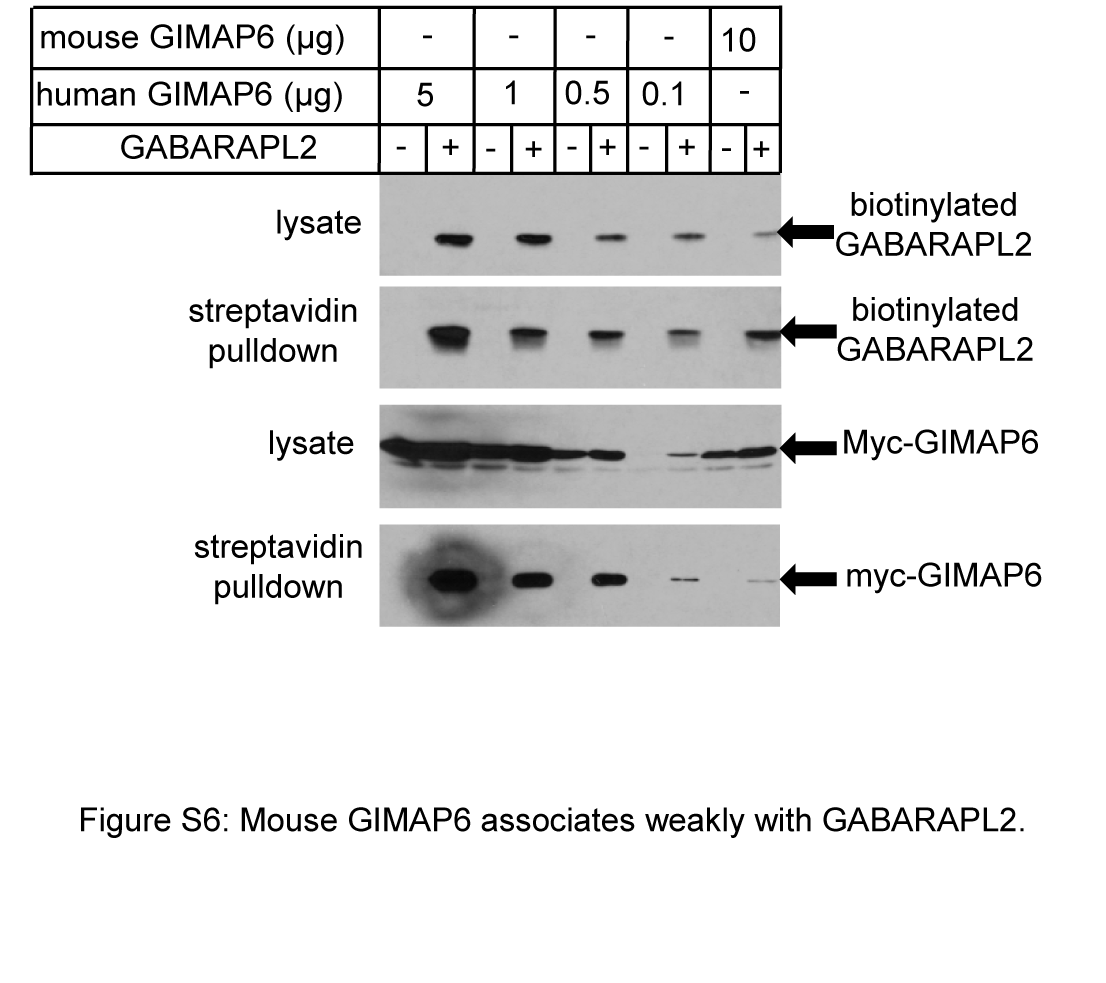

Supplement: Figure S6 — Mouse GIMAP6 associates weakly with GABARAPL2. HEK293T cells were transfected with myc-GIMAP6-encoding plasmids with or without GABARAPL2 in pcDNA3Biot1His6iresBirA, as indicated. Lysates were prepared, and biotinylated and associated proteins purified from the lysates using streptavidin-agarose. Western blots of the recovered proteins were probed with HRP-conjugated streptavidin (to show GABARAPL2) or a mouse monoclonal antibody (9E10) to the myc-tag followed by an HRP-conjugated goat anti-mouse IgG (to show GIMAP6). Western blots were developed using Immobilon ECL western blotting substrate. Interpretation of this experiment was complex as mouse GIMAP6 expressed only weakly in our transient assays compared with the human orthologue. However, co-immunoprecipitation of mouse GIMAP6 with GABARAPL2 was demonstrated in two independent experiments. (TIF) [file pone.0077782.s006.tif]

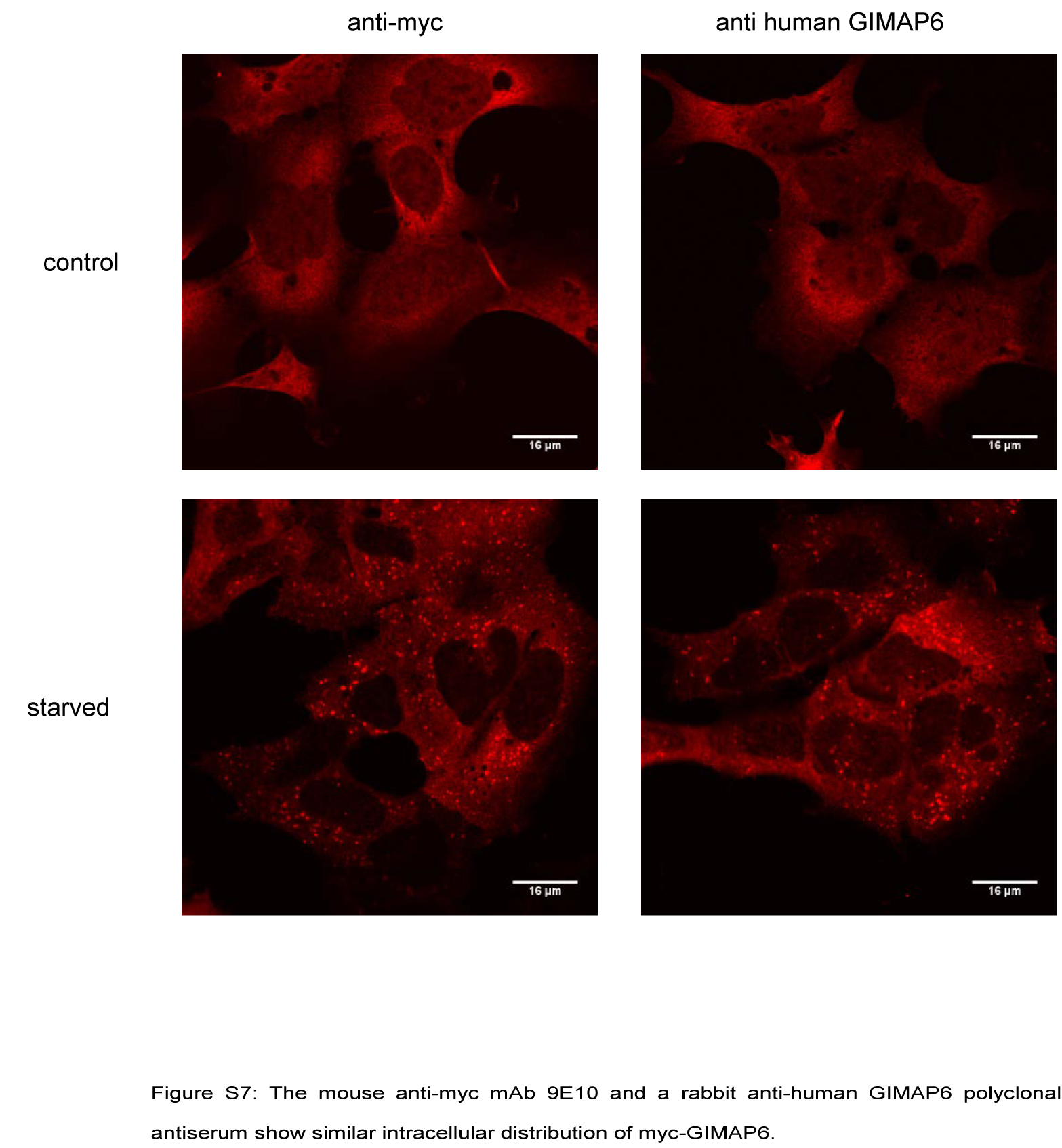

Supplement: Figure S7 — The mouse anti-myc mAb 9E10 and a rabbit anti-human GIMAP6 polyclonal antiserum show similar intracellular distribution of myc-GIMAP6. Myc-GIMAP6 HEK293 cells were either starved for 90 minutes or left untreated and were then processed for immunocytochemistry, using primary antibodies as indicated. The scale bars indicate 16 µm. The results shown are representative of three independent experiments. (TIF) [file pone.0077782.s007.tif]

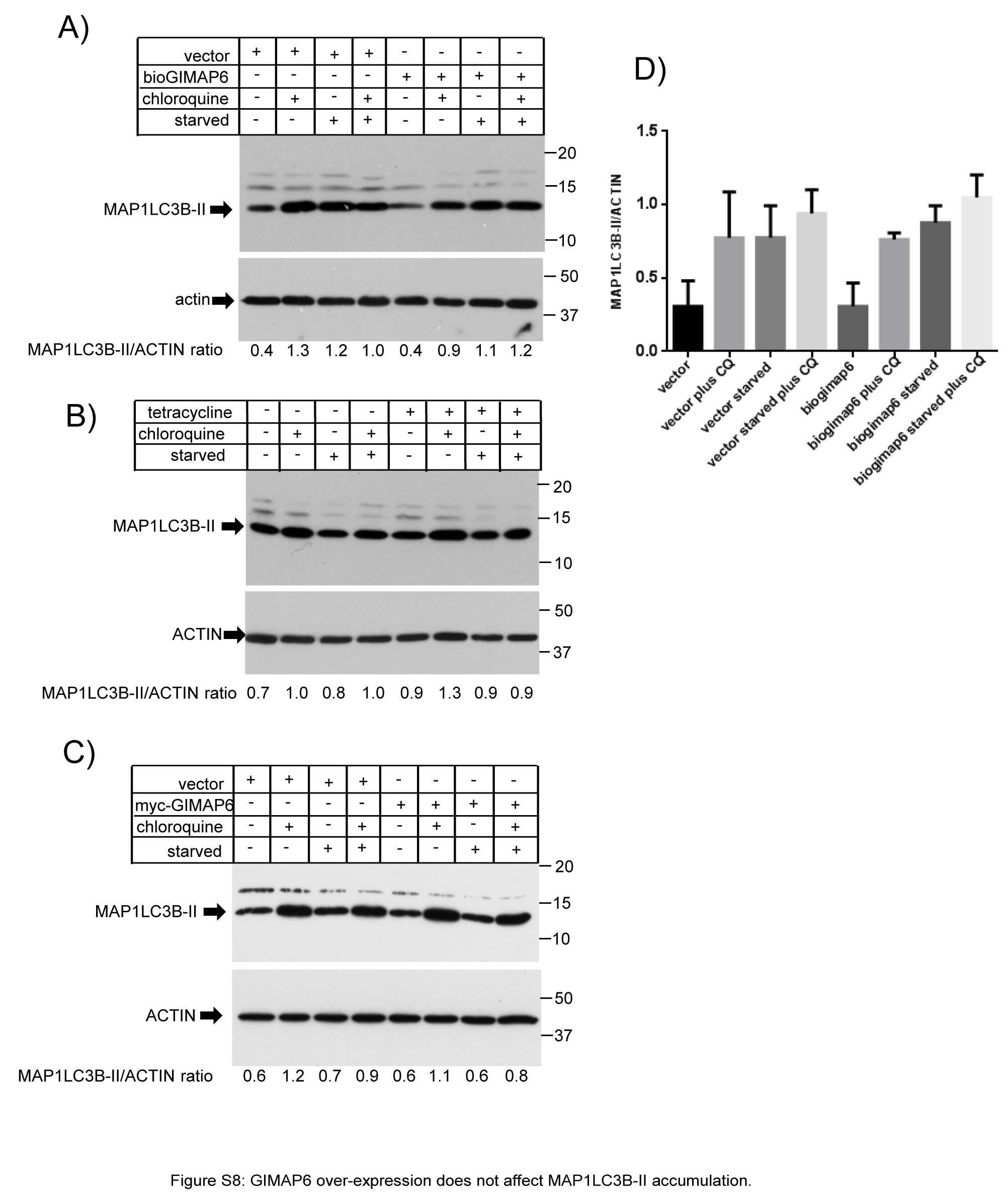

Supplement: Figure S8 — GIMAP6 over-expression does not affect MAP1LC3B-II accumulation. Cell lines (panel A – Biot-GIMAP6-His myc-BirA-Jurkat cell line or the corresponding parental cell line; panel B – myc GIMAP6 T-Rex HeLa cell line plus or minus tetracycline treatment; panel C – myc-GIMAP6 HEK293 cells or the corresponding vector control cells) were starved for 2 h (panels A and B) or 1.5h (panel C) or left untreated, with or without treatment with chloroquine as indicated. Cell lysates were prepared and analysed by SDS PAGE and Western blotting with antibodies to MAP1LC3B and ACTIN. Resulting X-ray films were scanned and images analysed using ImageJ software to determine MAP1LC3B-II/ACTIN ratios. Panel D – analysis of MAP1LC3B-II/ACTIN ratios for three experiments on the cells from Panel A. Results are shown as mean ± SD (n=3). The presence of chloroquine is indicated by CQ. One-way ANOVA indicated no significant statistical differences between treatments in the absence or presence of GIMAP6. (TIF) [file pone.0077782.s008.tif]

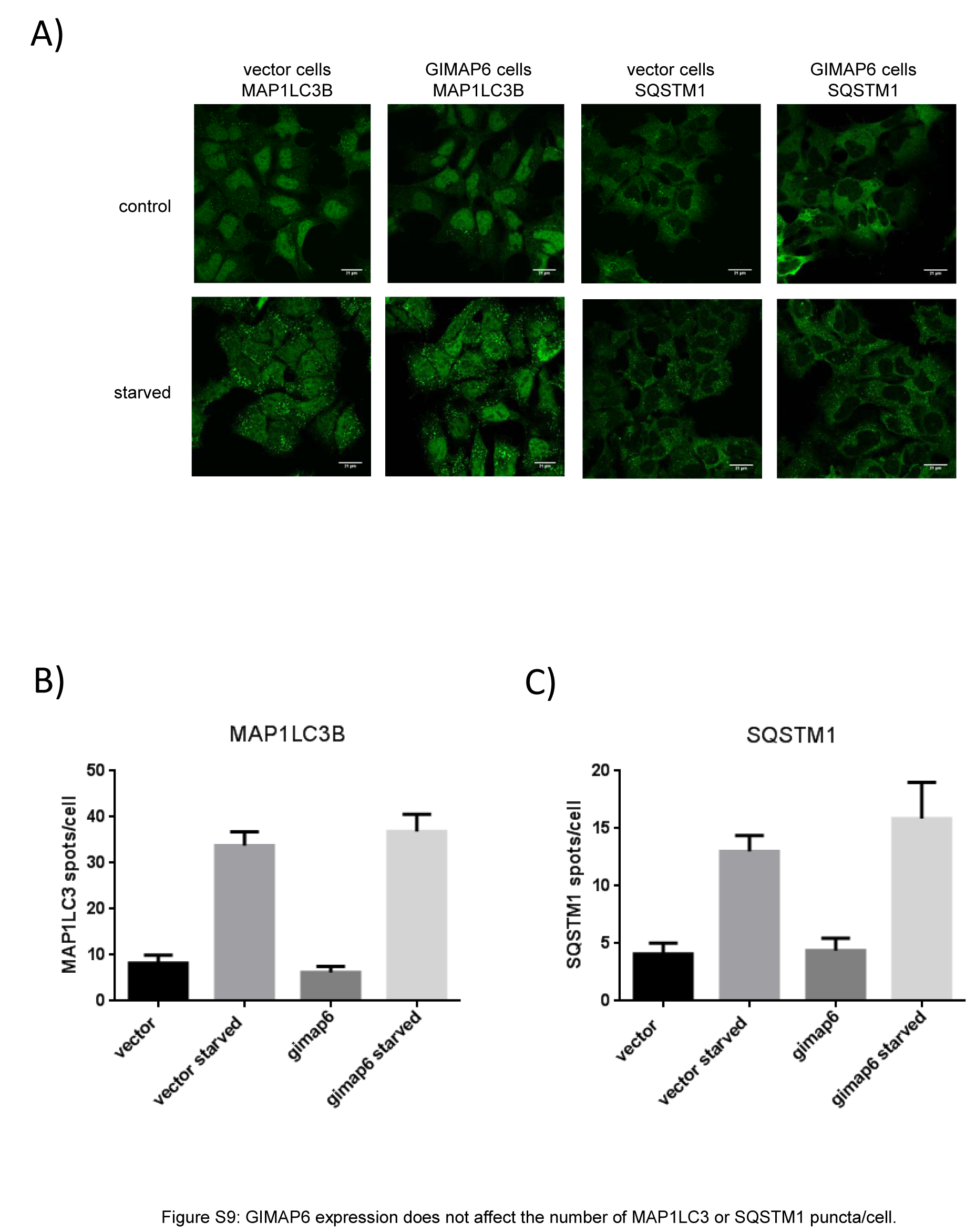

Supplement: Figure S9 — GIMAP6 expression does not affect the number of MAP1LC3 or SQSTM1 puncta/cell. myc-GIMAP6 HEK293 cells and the corresponding vector cells were either starved for 90 min or left untreated and subsequently immunocytochemically stained for MAP1LC3B or SQSTM1 as indicated. Typical images are shown in panel A. The scale bars represent 21 µm. Spots were counted (150-200 cells analysed for MAP1LC3B and 100-150 for SQSTM1 per condition). Analysis was performed using Imaris software. Results are presented as spots/cell ± SD – panel B MAP1LC3B; panel C SQSTM1. No significant difference was seen in the number of spots in the absence or presence of GIMAP6. Immunocytochemical results shown are representative of three independent experiments. (TIF) [file pone.0077782.s009.tif]
